# Corrosion of Hydrogen Storage Metal Alloy LaMm-Ni_4.1_Al_0.3_Mn_0.4_Co_0.45_ in the Aqueous Solutions of Alkali Metal Hydroxides

**DOI:** 10.3390/ma11122423

**Published:** 2018-11-30

**Authors:** Malgorzata Karwowska, Karol J. Fijalkowski, Andrzej A. Czerwiński

**Affiliations:** 1 Faculty of Chemistry, University of Warsaw, ul. Pasteura 1, 02-093 Warsaw, Poland; m.karwowska.chem@gmail.com (M.K.); aczerw@chem.uw.edu.pl (A.A.C.); 2 Centre of New Technologies, University of Warsaw, ul. Banacha 2c, 02-097 Warsaw, Poland; 3 Industrial Chemistry Research Institute, ul. Rydygiera 8, 01-793 Warsaw, Poland

**Keywords:** Ni-MH battery, AB_5_ alloy, hydrogen sorption, corrosion, limited volume electrode (LVE)

## Abstract

Corrosion of pristine AB_5_-type metal alloy LaMm-Ni_4.1_Al_0.3_Mn_0.4_Co_0.45_ in the aqueous solutions of alkali metal hydroxides of diverse composition and concentration was tested. Correlation was observed between the alloy corrosion intensity in various hydroxide solutions, and its electrochemical capacity in these electrolytes. Mm(OH)_3_, CoO(OH), and nickel metal aggregates were detected among the products of selective oxidation of the alloy. High intensity corrosion of the alloy was observed in RbOH and CsOH solutions leading to formation of ternary oxides at the surface of the alloy. Presence of rubidium and cesium ions in the electrolyte were found to create an additional driving force for lanthanides (La and Ce) to leave the lattice of the alloy, thus, enhancing its corrosion. Corrosion, together with mechanical degradation, were found to be the main reasons of deactivation of LaMm-Ni_4.1_Al_0.3_Mn_0.4_Co_0.45_ alloy upon elongated electrochemical treatment.

## 1. Introduction

Nickel-metal hydride batteries (Ni-MH) are still one of the most popular electrochemical power sources. Prior to the introduction of Li-ion batteries, Ni-MH batteries were widely used in mobile applications and hybrid electric vehicles [1,2,3,4]. The Ni-MH batteries, due to their high reliability and safety level, serve as a stationary uninterruptible power supply for various applications [5]. Nowadays, they are usually used to power high current demanding mobile devices, like digital cameras, flashlights, or electric vehicles, filling the gap between the heavy lead-acid batteries (starting batteries for combustion engines) and the light lithium-ion batteries (present in mobile electronic devices).

One of the most challenging features of the Ni-MH batteries is the loss of their overall capacity in a function of charging-discharging cycles being observed due to degradation and deactivation of anodic material over time [6]. Mechanical degradation of the active material, LaNi_5_-type hydrogen storage alloy, occurs due to the tension appearing in the lattice between charged and discharged regions, which have different molar volumes due to the insertion of hydrogen into the alloy, where the molar volume of hydrogenated LaNi_5_ alloy is up to 24% larger than the molar volume of dehydrogenated material [7]. The tension results in mechanical cracking of the material, eventually turning it into a powder, which causes its effective deactivation. Mechanical degradation can be overcome to some extent by the specific design of a cell and by modification of the alloy composition to minimize differences in unit cell volumes of charged and discharged materials [8].

Deactivation of the anodic material may occur also due to its chemical modification occurring over time. It was reported that LaNi_5_ alloy, as well as its doped and substituted analogues, undergoes various passivation processes during charging-discharging cycles in aqueous basic conditions. It was first reported by Willems that elongated electrochemical treatment of the LaNi_5_ electrode in 6 M KOH results in formation of a thick corrosion layer built of La(OH)_3_ needle-shape crystals covering the electrode surface [7]. Formation of lanthanides (mischmetals, Mm) hydroxides Mm(OH)_3_ was later observed and discussed several times [9,10,11,12,13,14]. Selective oxidation of AB_5_-type alloy towards rare earth metal oxides and hydroxides and metallic crystallites of the more noble metals was proposed [9]. The mechanism of formation of the thick porous oxide layer on the surface of the AB_5_ alloy grains, with the interface of the corrosion layer moving towards the center of the grains, was discussed [11].

Systematic characterization of the corrosion process of the AB_5_-type alloy, namely MmNi_3.55_Co_0.75_Mn_0.4_Al_0.3_, in concentrated KOH was performed by Maurel et al. [15]. It was reported that the ongoing decrease of the AB_5_-type alloy capacity by ca. 11% correlates with ca. 12% corrosion observed within the first 412 work cycles. It was observed that corrosion of the alloy occurs not only due to electrochemical treatment but also when soaking the electrode in concentrated KOH. Thus, presence of OH^−^ was discussed to be the key factor in the corrosion process, making corrosion of AB_5_ alloy thermodynamically favorable. Needle-shaped crystals of Mm(OH)_3_ were detected as the main corrosion product of the material. Also, octahedral crystals of MnO(OH)/Mn(OH)_2_ and spiky spheres of CoO(OH) were observed. Large metallic crystallites of more noble nickel and cobalt were found at the surface of the alloy as well. These crystalline corrosion products were located on top of a continuous corrosion layer comprising a mixture of nanocrystalline grains of metals (Ni, Co), oxides (Ni, Co)O, and mischmetal hydroxide Mm(OH)_3_. No solid corrosion products containing aluminum were observed at the anode due to the solubility of AlO^2–^ in basic conditions. Below the corrosion layer, a sublayer depleted from mischmetal was observed when investigating electrode cross-sections. In general, the continuous corrosion layers were observed to be ca. 200 nm thick, with the crystalline corrosion layer above reaching a 1500 nm thickness [15]. The corrosion mechanism was discussed to be highly dependent on basic conditions. Selective oxidation of the less noble elements with simultaneous reduction of the more noble ones could not be avoided thermodynamically in basic conditions [15]. Diffusion of lanthanides to the surface of the alloy was observed, resulting in the formation of a depleted sublayer rich in nickel and cobalt. It was also postulated that OH^−^ penetrates the alloy forming continuous corrosion layer of significant thickness [15]. Selective oxidation of the AB_5_-type alloy in basic conditions was confirmed by Morishita et al. [16].

Several ways to inhibit corrosion of AB_5_-type alloys were proposed and discussed. Addition of a small portion of Y_2_O_3_ to the anodic material or to the electrolyte was reported to improve electrode cycle lifetime by a factor of 2 [17]. Later it was discovered that Y_2_O_3_ incorporates into the continuous corrosion layer as Y(OH)_3_, reducing a driving force towards formation of Mm(OH)_3_, thus, reducing the corrosion rate of the electrode [18]. It was also found that substitution of cobalt with a more noble tin or copper decreases corrosion of the anodic material [19]. Recently, it was reported that substitution of La sites with nickel atoms can significantly enlarge the cycle lifetime of an anode, even up to 2415 cycles in comparison with the standard lifetime of ca. 500 cycles [20].

In this paper we present a study on corrosion and degradation of an AB_5_-type alloy occurring in contact with various alkali metal hydroxide solutions. Among the main goals of this study we show: (i) investigation of surface morphology changes of the AB_5_-type alloy after electrochemical experiments in alkali metal hydroxide solutions; (ii) identification of corrosion products formed at the surface of the AB_5_-type alloy, and (iii) determination of the optimum composition of the electrolyte allowing minimization of corrosion of the AB_5_-type alloy.

## 2. Materials and Methods

### 2.1. Working Electrode

LaMm-Ni_4.1_Al_0.3_Mn_0.4_Co_0.45_ hydrogen storage AB_5_-type alloy was used as a working electrode in all experiments. In our previous studies we have shown the general characteristic of this alloy at various temperatures (0–30 °C) and in various alkali metal electrolytes [21,22]. The alloy adopts a modified CaCu_5_ type crystal structure, related to that of pristine LaNi_5_ [23,24], adopting hexagonal P6/mmm unit cell: a = b = 5.0079(5) Å, c = 4.0521(4) Å, V = 88.007(16) Å^3^ (CSD number 427242) [21]. Average grain size is ca. 50 μm, spherical coefficient is ca. 1.146, while mass surface and volume surface are equal to 606 cm^2^/g and 1539 cm^2^/cm^3^, respectively [21], as shown in Table S1.

The limited volume electrode (LVE) approach was used to investigate LaMm-Ni_4.1_Al_0.3_Mn_0.4_Co_0.45_ electrode material free from additives and binding materials [25]. Binding materials were avoided (e.g., metal powders, graphite, polytetrafluoroethylene (PTFE), polyvinyl alcohol (PVA) since they can falsify the results of the electrochemical experiments [1,26,27,28,29]. LVEs were prepared by high pressure compression of investigated alloy between the two golden metal mesh (Good Fellow, nominal aperture 0.25 mm, 99.99% Au) under pressure of 20 MPa [21,22,30]. The designed and effective thickness of the electrodes of ca. 50 µm reflected the radius of the alloy particles. Gold mesh was selected as a matrix due to its electrochemical stability in a broad potential window, inertness to hydrogen, high electric conductivity, and good plastic properties, important when preparing stable electrode pallets. The LVE electrode was investigated by being placed inside a specially designed PTFE holder comprising polyethylene separators used in commercial Ni-MH batteries [21,22]. 

### 2.2. Electrolytes

All electrolytes were prepared using high purity compounds: LiOH (POCh, Gliwice, Poland), NaOH (POCh), KOH (POCh; Sigma Aldrich, St. Louis, MO, USA), RbOH (Sigma Aldrich), and CsOH (Sigma Aldrich). Two-step purified water was used: distilled and filtrated in reverse osmosis (Hydrolab, Straszyn, Poland), secondly deionized and UV irradiated (Millipore Simplicity 185, Millipore, Burlington, MA, US). 

### 2.3. Electrochemical Setup and Techniques

Electrochemical measurements were performed using PTFE vessels in a three-electrode system with an AB_5_-LVE working electrode, Hg|HgO reference electrode (6 M KOH, HYDROMET, Gliwice, Poland), and gold sheet counter electrode (Mint of Poland, Warsaw, Poland). All potentials presented in this paper refer to Hg|HgO (+0.14 V vs. NHE at 1 M NaOH). 

The AB_5_-LVE working electrodes were activated before the measurements using standard procedure comprising 50 cycles in a potential range of −1.1 V and −0.4 V at a rate of 2 mV/s. An AUTOLAB 30 analyzer (Autolab, Utrecht, The Netherlands) was used to perform chronoamperometric (CA), chronopotentiometric (CP), and voltammetric (CV) tests. A Lauda Proline 855 thermostat (Lauda, Lauda-Königshofen, Germany) with a KRYO51 bath was used for temperature control. 

### 2.4. Other Techniques

Pressure Composition Isotherms (PCI) were measured using a HTP1 Hiden Isochema (Warrington, UK). Very high purity (99.9999%) H_2_ and He gases were used. PCI measurements were done under pressure up to 90 atm in a temperature range of 35–150 °C. Activation of the investigated alloy was done under vacuum at 200 °C. Enegry Dispersive X-ray Spectroscopy (EDS) and Scanning Electron Microscopy (SEM) measurements were performed using a MERLIN Field Emission SEM (Zeiss, Oberkochen, Germany) to determine the composition of the studied alloy.

## 3. Results and Discussion

In our previous papers we have investigated LaMm-Ni_4.1_Al_0.3_Mn_0.4_Co_0.45_ as a hydrogen storage alloy for Ni-MH batteries, to optimize composition of the electrolyte at various temperatures in the range of 0–30 °C [21,22]. The highest electrochemical capacity, reaching almost 96% of the maximum hydrogen capacity (289.0 mAh/g, determined as the sorption of gaseous hydrogen), we observed in pure 6 M KOH at 30 °C (276.1 mAh/g) and in bimetallic 6 M LiOH/KOH at 30 °C (275.1 mAh/g), while performance in electrolytes containing NaOH was slightly lower. At the same time, we noticed significantly lower capacities when using bimetallic electrolytes containing RbOH and CsOH, as shown in Figures S5 and S6. 

Simultaneously, we observed corrosion and degradation of the working electrode ongoing when treated in basic aqueous electrolytes. In general, the longer the treatment of the electrode, the larger the amount of corrosion products was found for each electrolyte investigated. Intensity of the observed corrosion correlates well with the capacity of the alloy in respective electrolytes determined previously. We observed also a general decrease of capacity of the alloy over time after numerous charging/discharging cycles, which might be caused by a combination of two factors: corrosion in basic condition [15] and mechanical cracking of the alloy due to tension between charged and discharged alloy phases [8]. We confirmed this possibility, proving brittleness of the alloy upon hydrogenation and dehydrogenation using a PCI approach, as shown in Figure S7 and Table S2.

### 3.1. Corrosion in LiOH, NaOH, and KOH Solutions

**Figure 1:**
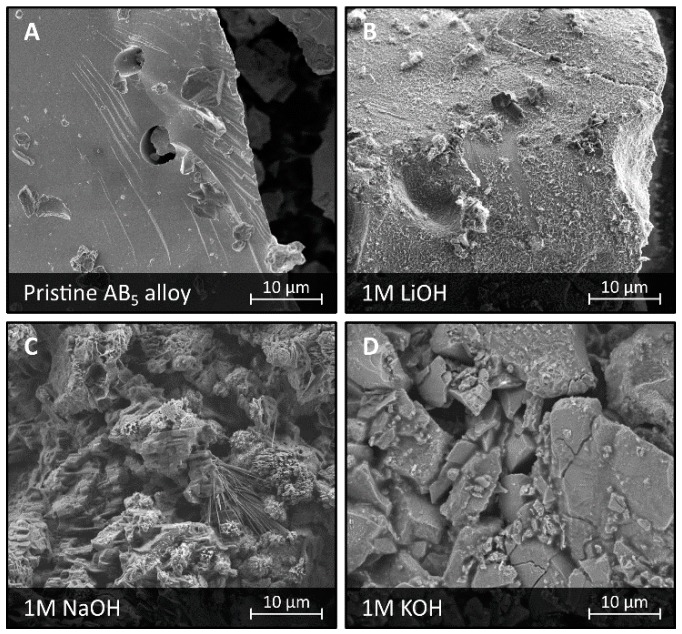
SEM images of a surface of LaMm-Ni_4.1_Al_0.3_Mn_0.4_Co_0.45_ alloy not subjected to any treatment (**A**), and after electrochemical treatment in 1 M LiOH (**B**), 1 M NaOH (**C**), and 1 M KOH (**D**).

The surface of LaMm-Ni_4.1_Al_0.3_Mn_0.4_Co_0.45_ alloy was investigated with SEM imaging after electrochemical treatment in electrolytes containing light alkali metal hydroxides, as shown in Figure 1A–D. The alloy was activated with a standard CV procedure and subjected to several cycles of charging/discharging before the surface investigation. In general, rather minor surface modifications were observed after electrochemical treatment in light alkali metal hydroxides. Only small and medium size needle-shape crystals, not fully covering the surface of the alloy, were found. Shape, size, and composition determined by EDS allowed us to identify these crystals as Mm(OH)_3_, as shown in Figures S8 and S9. No other crystalline corrosion formations nor amorphous corrosion structures were found. 

The largest Mm(OH)_3_ needle-shape crystals, of a length reaching ca. 10 μm, were observed at the surface of the alloy treated in NaOH electrolytes (1 M NaOH, 6 M NaOH). We found also that treatment in NaOH results in the relatively largest coverage of the alloy surface by Mm(OH)_3_ among all the light metal hydroxide solutions. Corrosion in LiOH and KOH electrolytes (1 M LiOH, 1 M KOH, 6 M KOH) was ongoing with significantly lower intensity resulting in lower surface coverage with Mm(OH)_3_ needles. These observations are in very good agreement with the results of the electrochemical test showing the largest capacities of the alloy observed in 6 M LiOH/KOH and 6 M KOH, with slightly lower capacities at 6 M NaOH/KOH [22].

### 3.2. Corrosion in RbOH Solutions

**Figure 2:**
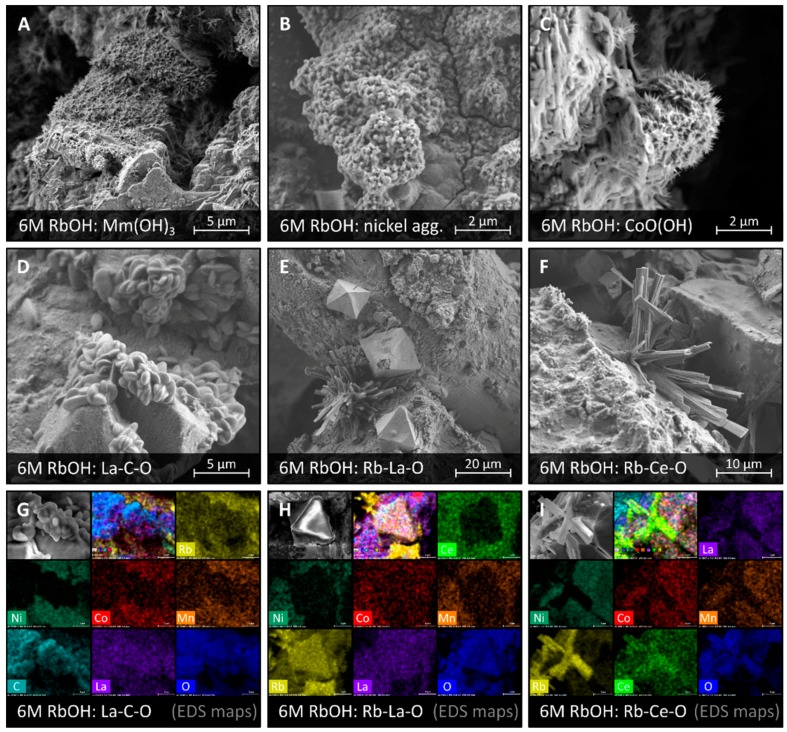
SEM images of a surface of LaMm-Ni_4.1_Al_0.3_Mn_0.4_Co_0.45_ alloy after electrochemical treatment in 6 M RbOH showing surface formations of: Mm(OH)_3_ (**A**), nickel aggregations (**B**), CoO(OH) (**C**), La-C-O (**D**), Rb-La-O (**E**), and Rb-Ce-O (**F**). EDS maps showing elemental distribution in the areas where corrosive surface formations were found: La-C-O (**G**), Rb-La-O (**H**), and Rb-Ce-O (**I**).

The surface of LaMm-Ni_4.1_Al_0.3_Mn_0.4_Co_0.45_ alloy treated in RbOH solutions (1 M RbOH, 6 M RbOH) was found to be covered with a nonuniform corrosion layer containing various crystalline products of different morphology and elemental composition, leaving large parts of the surface uncovered. The majority of them were forming a continuous corrosion layer comprising lanthanide hydroxides, Mm(OH)_3_, the needle-shaped crystals of Mm(OH)_3_, and particles of metallic nickel, as shown in Figure 2A,B, Figures S10 and S11 and Table S4. Also found were crystals of CoO(OH) in a shape of small spiky spheres (ca. 3 μm) covered with short sharp needles, as shown in Figure 2C. Similar corrosion products were described by Maurel et al. [15].

We have discovered the three new crystalline corrosion products, as shown in Figure 2D–F, not described before to appear at the surface of the AB_5_-type alloy. Attempts of their identification was performed using elemental EDS analysis, as shown in Figure 2G–I, Figures S12–S19 and Tables S5–S8, supported by a search in a crystallographic database [31]. Unfortunately, our attempts to isolate these corrosion products and investigate them with X-ray diffraction were unsuccessful due to the small size of their crystals and relatively low number of well-formed corrosion sites.

Round-shaped small crystals most likely might be one of the hydrogenated lanthanum carbonates or oxalates, as shown in Figure 2D,G. The EDS elemental analysis showed high content of lanthanum, oxygen, and carbon within the crystals, while concentration of all other possible elements was much lower: Ni, Co, Mn, Al, Ce, Nd, Pr, Au, as shown in Figures S5 and S6 and Table S5. Ten crystalline La-C-O compounds were found in the Inorganic Crystal Structure Database (ICSD) [31], all of which were lanthanum carbonates and oxalates at various levels of hydration, as shown in Table S10. It is impossible to judge which of those compounds were observed in our samples. La-C-O crystals were relatively small, not exceeding 2 μm in diameter, and they were appearing in a larger formation of 80–100 crystals.

Crystals in the shape of bipyramids are most likely rubidium-lanthanum oxide, as shown in Figure 2E,H. EDS elemental analysis showed simultaneous high content of rubidium, lanthanum, and oxygen within the crystals, with depletion of all other elements possible to be found in this sample: Ni, Co, Mn, Al, Ce, Nd, Pr, Au, as shown in Figures S14 and S15 and Table S6. RbLaO_2_ was the only compound found in the ICSD crystallographic database, as shown in Table S10 [31], to contain exclusively rubidium, lanthanum, and oxygen [32]. Rb-La-O crystals observed were well-formed, having 15–20 μm in diameter, and appearing in groups of 5–10.

The elongated rectangular crystals are most likely rubidium-cerium oxide, as shown in Figure 2F,I. High concentration of rubidium, cerium, and oxygen was detected within the crystals with EDS, as shown in Figures S16 and S17 and Table S7. Rb_2_CeO_3_ was the only compound containing Rb-Ce-O elements [33] found in the crystallographic database [31]. Purity of these crystals may result from inaccessibility of the +IV oxidation state by other lanthanides than cerium. The observed crystals were of various sizes starting from 2–3 μm in length, fully stuck to the surface, up to the crystals of 10–16 μm long, growing perpendicularly to the surface. Most often, the larger formations of Rb-Ce-O were found comprising 20–30 crystals growing from a common broader base.

Presence of rubidium containing crystals as products of corrosion of the AB_5_-type alloy prove one of the corrosion mechanisms proposed by Maurel et al. [15], namely the mechanism of lanthanide diffusion in the solid state towards the surface to react with OH^−^ and form Mm(OH)_3_ crystals. Our study shows that presence of rubidium cations in the electrolyte enhance the intensity of the AB_5_ alloy corrosion by creation of an additional driving force for lanthanides (La and Ce) to leave the lattice and create Rb-La-O and Rb-Ce-O crystals. Spontaneous formation of these specific corrosion sites prevented, however, uniform coverage of the alloy by a corrosion layer, leaving large uncovered areas of the alloy allowing relatively high capacity comparable with capacity in the NaOH electrolyte.

As a consequence of formation of new and separate lanthanum and cerium rich phases, the alloy and the corrosion layer are depleted from these elements, and thus, are enriched with all other constituent elements (Ni, Co, Mn, Al, Nd, Pr). It was confirmed by EDS analysis of selected areas of the alloy’s surface, as shown in Figures S18 and S19 and Table S8, where corrosive formations were found.

### 3.3. Corrosion in CsOH Solutions

**Figure 3:**
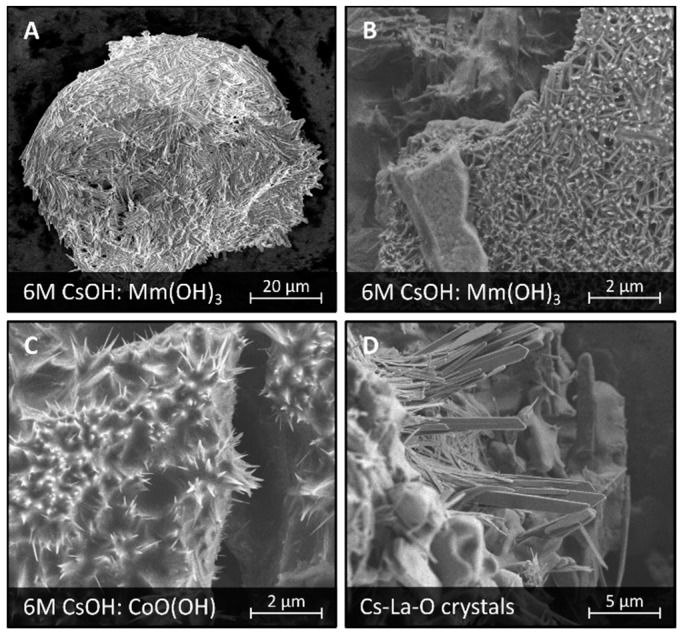
SEM images of a surface of LaMm-Ni_4.1_Al_0.3_Mn_0.4_Co_0.45_ alloy after electrochemical treatment at 6 M CsOH showing surface formations of: Mm(OH)_3_ (**A**,**B**), CoO(OH) (**C**), and Cs-La-O (**D**).

The surface of LaMm–Ni_4.1_Al_0.3_Mn_0.4_Co_0.45_ alloy was covered with the products of its corrosion after treatment in CsOH solutions (1 M CsOH, 6 M CsOH). The surface was mostly covered by a thick continuous corrosion layer of crystalline Mm(OH)_3_, as shown in Figure 3A,B, and small spiky crystals of CoO(OH), as shown in Figure 3C, described before [15]. However, areas of uncovered surface were noticed. 

We observed one corrosion product which was not reported earlier in similar systems, as shown in Figure 3D. This compound forms elongated crystals of a morphology reminiscent of the Rb-Ce-O crystals described above. However, the EDS analysis showed that the only elements present in these crystals are cesium, oxygen, and lanthanum (instead of cerium), with depletion of all other elements which might be present in this sample: Ni, Co, Mn, Al, Ce, Nd, Pr, Au, as shown in Figure S20 and Table S9. Thus, the elongated crystals are most likely cesium-lanthanum oxide. No Cs-La-O compounds were found in the ICSD, as shown in Table S10 [31], however, one compound of this composition, mainly CsLaO_2_, was previously mentioned in the literature [34]. Cs-La-O crystals having an average length of 5–15 μm, appear in large formations of 20–30 crystals growing from a common base.

CsOH electrolyte enhances the rate of corrosion of the AB_5_ alloy, similarly to RbOH, but leading to more uniform surface coverage resulting in a capacity decrease of the alloy. It is worth mentioning that CsOH is a very aggressive medium, which causes corrosion of various materials, including glass. Cesium is able to form crystalline ternary compounds with lanthanum and oxygen, which provides an additional driving force for lanthanum to migrate to the surface of the alloy.

### 3.4. Corrosion in Bimetallic Mixed Electrolytes Based on KOH

**Figure 4:**
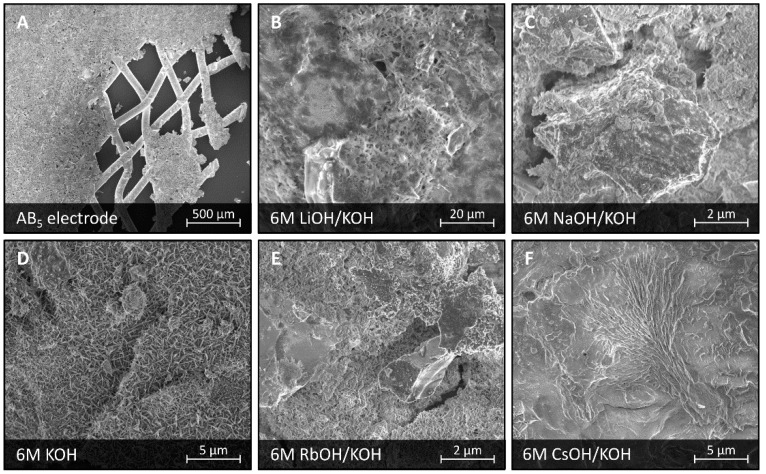
SEM images of a surface of LaMm-Ni_4.1_Al_0.3_Mn_0.4_Co_0.45_ alloy not subjected to any treatment (**A**), and after electrochemical treatment at 6 M electrolytes: 6 M LiOH/KOH (**B**), 6 M NaOH/KOH (**C**), 6 M KOH (**D**), 6 M RbOH/KOH (**E**), and 6 M CsOH/KOH (**F**).

Surprisingly, corrosion of LaMm-Ni_4.1_Al_0.3_Mn_0.4_Co_0.45_ alloy, electrochemically treated in 6 M mixed electrolytes containing 4 M KOH and 2 M additive of another component (LiOH, NaOH, RbOH, or CsOH), is much less dependent on composition of the electrolyte than in the case of single component electrolytes, and it is similar to corrosion occurring solely in 6 M KOH, as shown in Figure 4. In all cases of pristine 6 M KOH and bimetallic 6 M MOH/KOH electrolytes, corrosion goes in a direction of selective oxidation of lanthanides, as described before [15]. No other large corrosion formations, even in electrolytes containing RbOH and CsOH, were observed, as shown in Figure 4E,F. 

Differences between corrosion of the AB_5_-type alloy in 6 M electrolytes of different composition occur in morphology of the corrosion layer covering the surface of the alloy. In pristine 6 M KOH, the corrosion layer was relatively thin and formed of separate needle-shaped Mm(OH)_3_ crystals, not fully covering the surface. A similar layer was observed in 6 M LiOH/KOH and 6 M NaOH/KOH. In contrast, the corrosion layer in 6 M RbOH/KOH and 6 M CsOH/KOH seems to be rather of a continuous morphology and large thickness, uniformly covering the full surface. These differences in morphology and degree of surface blockage by continuous layers might explain the much lower capacities of the alloy observed in RbOH/KOH and CsOH/KOH bimetallic electrolytes in comparison with solutions containing lighter alkali metal hydroxides. 

Rubidium and cesium cations present in the KOH-based electrolytes seem to enhance formation of a thick corrosion layer uniformly covering the surface of the alloy. Even though large crystalline formations of rubidium and cesium containing corrosion products could not be observed, it is very likely that small crystallites of Rb-La-O, Rb-Ce-O, and Cs-La-O were formed and fully incorporated in the corrosion layer. This would explain the larger intensity of corrosion, larger thickness of the corrosion layer, and larger overall surface blockage observed in RbOH/KOH and CsOH/KOH electrolytes, causing significant decrease of the alloy’s capacity.

## 4. Summary and Conclusions

We showed a detailed study of the corrosion process of hydrogen storage AB_5_-type metal alloy LaMm-Ni_4.1_Al_0.3_Mn_0.4_Co_0.45_ in alkali metal hydroxide solutions. We observed characteristic products of selective oxidation of AB_5_ alloys described in the literature [9,11,15]: formations of Mm(OH)_3_ needle-shape crystals and nickel metal aggregates. 

We have observed a very good correlation between the intensity of corrosion, morphology, and thickness of the corrosion layer, and capacity of the alloy determined in respective electrolytes. The least intensive corrosion resulting in formation of the thinnest corrosion layer of Mm(OH)_3_ was observed at the surface of the electrodes treated in LiOH, NaOH, and KOH containing solutions, where we observed the highest capacities of the investigated AB_5_ alloy [21,22]. On the other hand, the largest corrosion was observed when the alloy was treated in RbOH and CsOH, where the lowest capacities were determined [21,22].

The enhanced corrosion observed in RbOH and CsOH containing electrolytes can be explained by the large affinity of rubidium and cesium cations towards the constituent elements of the investigated AB_5_ alloy: lanthanum and cerium. Presence of rubidium and cesium cations in the electrolyte creates an additional driving force for lanthanides (La and Ce) to leave the lattice of the alloy to form ternary compounds (probably: RbLaO_2_, Rb_2_CeO_3_, CsLaO_2_) at its surface. Corrosion products containing rubidium and cesium are usually grouped in larger aggregates suggesting depletion of the alloy from lanthanum and cerium in the vicinity of these regions when formed in pristine RbOH and CsOH. When corrosion occurs in bimetallic RbOH/KOH and CsOH/KOH electrolytes, a thick and uniform corrosion layer is formed (much thicker than in pristine KOH, RbOH, and CsOH), probably containing small crystallites of Rb-La-O, Rb-Ce-O, or Cs-La-O. 

The results presented in this study suggest that corrosion processes occurring at the surface of the AB_5_-type alloy in basic conditions, together with its mechanical degradation caused by numerous cycles of charging and discharging, might be the main reasons of deactivation of AB_5_-type electrodes and observed decreasing performance of Ni-MH batteries.

Optimization of the electrolyte composition may play an important role when enhancing overall performance of Ni-MH batteries. To avoid rapid corrosion and selective oxidation of the alloy, which thermodynamically cannot be avoided, the electrolyte should not contain elements with high affinity to lanthanides. When selecting components of the electrolyte among alkali metal hydroxides, one should avoid rubidium and cesium containing solutions since these elements form ternary oxides with constituent elements of the alloy (lanthanum and cerium). 

We have shown that use of basic electrolytes based on KOH with smaller additions of LiOH and NaOH can lead to enhanced performance of AB_5_ electrodes in comparison to their performance in pristine KOH. 

The presented electrochemical study may be important in view of designing new electrolytes to be used in Ni-MH batteries, however, further work on the optimization of electrolyte is needed.

## Supplementary Materials

The following are available online at http://www.mdpi.com/1996-1944/11/12/2423/s1, Figure S1. SEM images of LaMmNi_4.1_Al_0.2_Mn_0.4_Co_0.45_ alloy particles, Figure S2. Comparison of X-ray powder diffractograms of investigated LaMmNi_4.1_Al_0.2_Mn_0.4_Co_0.45_ alloy, collected with using λ = 1.78901 Å [21] and pristine LaNi_5_ alloy [23], Figure S3. EDS images of a surface of pristine LaMmNi_4.1_Al_0.2_Mn_0.4_Co_0.45_ alloy with mapping of elemental distribution of lanthanum, nickel, cerium, cobalt, manganese, and aluminum, Figure S4. EDS spectrum of a surface of pristine LaMmNi_4.1_Al_0.2_Mn_0.4_Co_0.45_ alloy not subjected to electrochemical treatment [21], Table S1. Results of EDS elemental analysis of pristine LaMmNi_4.1_Al_0.2_Mn_0.4_Co_0.45_ alloy not subjected to electrochemical treatment [21], Figure S5. Electrochemical capacity of LaMm–Ni_4.1_Al_0.3_Mn_0.4_Co_0.45_ alloy as a function of composition of 1 M and 6 M MOH (M = Li,Na,K,Rb,Cs) solutions at temperatures in the range 0–30 °C in a series of decreasing temperatures [21], Figure S6. Electrochemical capacity of LaMm–Ni_4.1_Al_0.3_Mn_0.4_Co_0.45_ alloy as a function of composition of 6 M MOH/KOH (M = Li–Cs) solutions at temperatures in the range 0–30 °C in a series of decreasing temperature [21], Figure S7. Distribution of the particle size of the LaMmNi_4.1_Al_0.2_Mn_0.4_Co_0.45_ alloy: pristine alloy (bottom), hydrogenated alloy (middle), and alloy after 5 cycles of hydrogenation with gaseous H_2_ (top). Particle size [μm] showed in a function of volume fraction Bv [%]. Percentile values of 10%, 50%, and 90% of the population exposed with grey fields. Sample population e 200,000 particles. Spectrum of pristine alloy were shown by us before [21], Table S2. Results of granulometric determination of particle size distribution of the LaMmNi_4.1_Al_0.2_Mn_0.4_Co_0.45_ alloy: pristine alloy, hydrogenated alloy, and alloy after 5 cycles of hydrogenation with gaseous H_2_. Parameters of pristine alloy were shown by us before [21], Figure S8. EDS surface images of LaMmNi_4.1_Al_0.2_Mn_0.4_Co_0.45_ alloy electrochemically treated in 6 M KOH at 30 °C. Mapping of elemental distribution of rubidium, lanthanum, praseodymium, aluminum, nickel, manganese, and cerium, Figure S9. EDS spectrum of the corrosive layer at the surface of LaMmNi_4.1_Al_0.2_Mn_0.4_Co_0.45_ alloy electrochemically treated in 6 M KOH at 30 °C, Table S3. Results of EDS elemental analysis of the corrosive layer at the surface of LaMmNi_4.1_Al_0.2_Mn_0.4_Co_0.45_ alloy electrochemically treated in 6 M KOH at 30 °C, Figure S10. EDS surface images of LaMmNi_4.1_Al_0.2_Mn_0.4_Co_0.45_ alloy electrochemically treated in 6 M RbOH at 30 °C, with visible nickel metal particle. Mapping of elemental distribution of rubidium, lanthanum, praseodymium, aluminum, nickel, manganese, and cerium, Figure S11. EDS spectrum of the area of nickel metal particle found at the surface of LaMmNi_4.1_Al_0.2_Mn_0.4_Co_0.45_ alloy electrochemically treated in 6 M RbOH at 30 °C, Table S4. Results of EDS elemental analysis of nickel metal particle found at the surface of LaMmNi_4.1_Al_0.2_Mn_0.4_Co_0.45_ alloy electrochemically treated in 6 M RbOH at 30 °C, Figure S12. EDS surface images of LaMmNi_4.1_Al_0.2_Mn_0.4_Co_0.45_ alloy electrochemically treated in 6 M RbOH at 30 °C, with visible La-C-O-H round-shaped crystals. Mapping of elemental distribution of rubidium, nickel, cobalt, manganese, carbon, oxygen, and lanthanum, Figure S13. EDS spectrum of the area of La-C-O crystals found at the surface of LaMmNi_4.1_Al_0.2_Mn_0.4_Co_0.45_ alloy electrochemically treated in 6 M RbOH at 30 °C, Table S5. Results of EDS elemental analysis of the area of La-C-O crystals found at the surface of LaMmNi_4.1_Al_0.2_Mn_0.4_Co_0.45_ alloy electrochemically treated in 6 M RbOH at 30 °C, Figure S14. EDS surface images of LaMmNi_4.1_Al_0.2_Mn_0.4_Co_0.45_ alloy electrochemically treated in 6 M RbOH at 30 °C, with visible Rb-La-O bipyramidal-shaped crystals. Mapping of elemental distribution of nickel, cerium, manganese, cobalt, rubidium, lanthanum, and oxygen, Figure S15. EDS spectrum of the area of Rb-La-O crystals found at the surface of LaMmNi_4.1_Al_0.2_Mn_0.4_Co_0.45_ alloy electrochemically treated in 6 M RbOH at 30 °C, Table S6. Results of EDS elemental analysis of the area of Rb-La-O crystals found at the surface of LaMmNi_4.1_Al_0.2_Mn_0.4_Co_0.45_ alloy electrochemically treated in 6 M RbOH at 30 °C, Figure S16. EDS surface images of LaMmNi_4.1_Al_0.2_Mn_0.4_Co_0.45_ alloy electrochemically treated in 6 M RbOH at 30 °C, with visible Rb-Ce-O bipyramidal-shaped crystals. Mapping of elemental distribution of cobalt, nickel, lanthanum, manganese, rubidium, cerium, and oxygen, Figure S17. EDS spectrum of the area of Rb-Ce-O crystals found at the surface of LaMmNi_4.1_Al_0.2_Mn_0.4_Co_0.45_ alloy electrochemically treated in 6 M RbOH at 30 °C, Table S7. Results of EDS elemental analysis of the area of Rb-Ce-O crystals found at the surface of LaMmNi_4.1_Al_0.2_Mn_0.4_Co_0.45_ alloy electrochemically treated in 6 M RbOH at 30 °C, Figure S18. EDS surface images of LaMmNi_4.1_Al_0.2_Mn_0.4_Co_0.45_ alloy electrochemically treated in 6 M RbOH at 30 °C with visible disproportionation of more-noble and less-noble elements. Mapping of elemental distribution of aluminum, nickel, cobalt, manganese, lanthanum, cerium, neodymium, rubidium, oxygen, and carbon, Figure S19. EDS spectrum of the surface of LaMmNi_4.1_Al_0.2_Mn_0.4_Co_0.45_ alloy electrochemically treated in 6 M RbOH at 30 °C, Table S8. Results of EDS elemental analysis of the surface of LaMmNi_4.1_Al_0.2_Mn_0.4_Co_0.45_ alloy electrochemically treated in 6 M RbOH at 30 °C, Figure S20. SEM image of Cs-La-O crystals at the surface of LaMmNi_4.1_Al_0.2_Mn_0.4_Co_0.45_ alloy electrochemically treated in 1M CsOH at 20 °C (left) and EDS spectrum of these crystals (right), Table S9. Results of EDS elemental analysis of Cs-La-O crystals at the surface of LaMmNi_4.1_Al_0.2_Mn_0.4_Co_0.45_ alloy electrochemically treated in 1 M CsOH, Table S10. Summary of search in Inorganic Crystal Structure Database by Fachinformationszentrum Karlsruhe for known compounds comprising elements detected during EDS investigation of corrosion products observed after treatment of LaMmNi_4.1_Al_0.2_Mn_0.4_Co_0.45_ alloy in RbOH and CsOH. Access in September 2018.
