# Supplementary material for: Corrosion of Hydrogen Storage Metal Alloy LaMm-Ni4.1Al0.3Mn0.4Co0.45 in the Aqueous Solutions of Alkali Metal Hydroxides
Source: Materials (Basel). 2018 Nov 30;11(12):2423. doi: 10.3390/ma11122423 (PMC6316957; doi:10.3390/ma11122423)
Supplement: Supplementary file 1 [file materials-11-02423-s001.pdf]

materials

Supplementary Materials: Corrosion of Hydrogen Storage Metal Alloy LaMm-$Ni_{4.1}Al_{0.3}Mn_{0.4}Co_{0.45}$ in Alkali Metals Electrolytes

Malgorzata Karwowska, Karol J. Fijalkowski and Andrzej A. Czerwiński

Table of Contents:

1. LaMm-$Ni_{4.1}Al_{0.3}Mn_{0.4}Co_{0.45}$ alloy
- SEM images of the alloy particles
- Powder X-ray diffraction patterns of the alloy
- EDS images of the surface of the alloy
- EDS spectrum and numeric results of EDS analysis of the surface of the alloy

2. Electrochemical capacity of LaMm-$Ni_{4.1}Al_{0.3}Mn_{0.4}Co_{0.45}$ alloy in alkaline solutions
- Electrochemical capacity of the alloy in 1M and 6M MOH solutions
- Electrochemical capacity of the alloy in 6M MOH/KOH solutions

3. Mechanical degradation of LaMm-$Ni_{4.1}Al_{0.3}Mn_{0.4}Co_{0.45}$ alloy after gaseous H_2 treatment
- Particle size distribution spectra of the alloy upon sorption of gaseous hydrogen
- Particle size distribution data of the alloy upon sorption of gaseous hydrogen

4. EDS images of surface formations observed after treatment in KOH solution
- EDS images showing needle-shaped crystals of $Mm(OH)_3$
- EDS spectrum and numeric results of EDS analysis of $Mm(OH)_3$ crystals

5. EDS images of surface formations observed after treatment in RbOH solution
- EDS images showing nickel particles
- EDS spectrum and numeric results of EDS analysis of nickel particles
- EDS images showing La-C-O-H crystals
- EDS spectrum and numeric results of EDS analysis of La-C-O-H crystals
- EDS images showing Rb-La-O crystals
- EDS spectrum and numeric results of EDS analysis of Rb-La-O crystals
- EDS images showing Rb-Ce-O crystals
- EDS spectrum and numeric results of EDS analysis of Rb-Ce-O crystals
- EDS images showing disproportionation of the alloy composition
- EDS spectrum and numeric results of EDS analysis of the surface of the alloy

6. EDS images of surface formations observed after treatment in CsOH solution
- SEM images of the surface of the alloy showing Cs-La-O crystals
- EDS spectrum and numeric results of EDS analysis of Cs-La-O crystals

7. Results of search in Inorganic Crystal Structure Database by FIZ Karlsruhe
- Results of search of La-C-O, Rb-La-O, Rb-Ce-O and Cs-La-O compounds in ICSD

1. LaMm-$Ni_{4.1}Al_{0.3}Mn_{0.4}Co_{0.45}$ alloy [1/3]

SEM images of LaMm$Ni_{4.1}Al_{0.2}Mn_{0.4}Co_{0.45}$ alloy particles

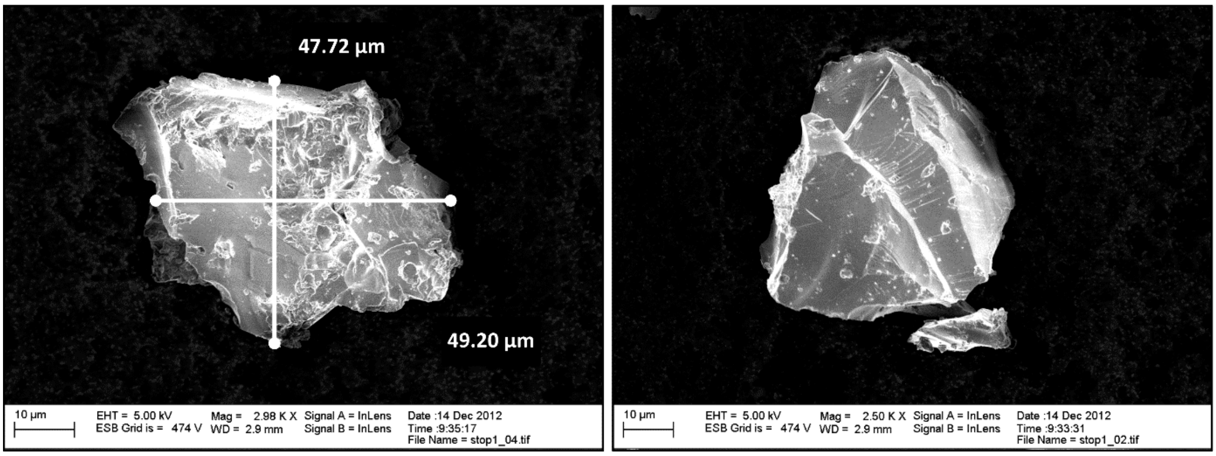

Figure S1. SEM images of LaMm$Ni_{4.1}Al_{0.2}Mn_{0.4}Co_{0.45}$ alloy particles.

Comparison of X-ray powder diffractograms of LaMm$Ni_{4.1}Al_{0.2}Mn_{0.4}Co_{0.45}$ alloy and pristine $LaNi_5$ alloy

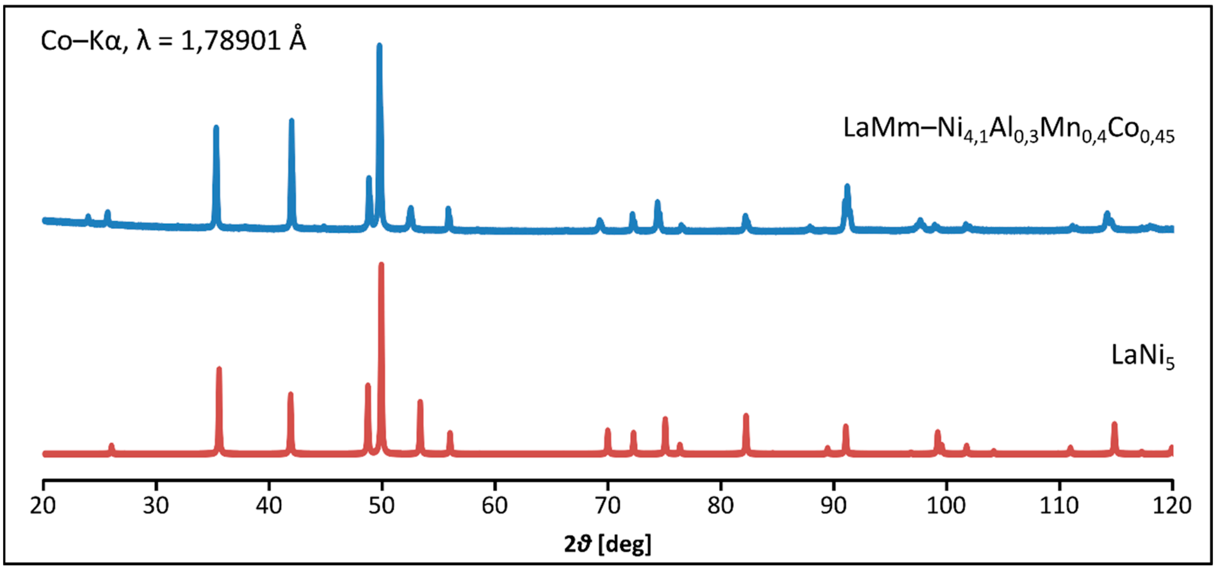

Figure S2. Comparison of X-ray powder diffractograms of investigated LaMm$Ni_{4.1}Al_{0.2}Mn_{0.4}Co_{0.45}$ alloy, collected with using λ = 1.78901 Å [M. Karwowska *et al.*, *J. Power Sources*, 263 (**2014**) 304] and pristine $LaNi_5$ alloy [H.N. Nowotny, *Z. Metallkd.* 34 (**1942**) 247].

1. LaMm-$Ni_{4.1}Al_{0.3}Mn_{0.4}Co_{0.45}$ alloy [2/3]

EDS images of a surface of pristine $LaMmNi_{4.1}Al_{0.2}Mn_{0.4}Co_{0.45}$ alloy

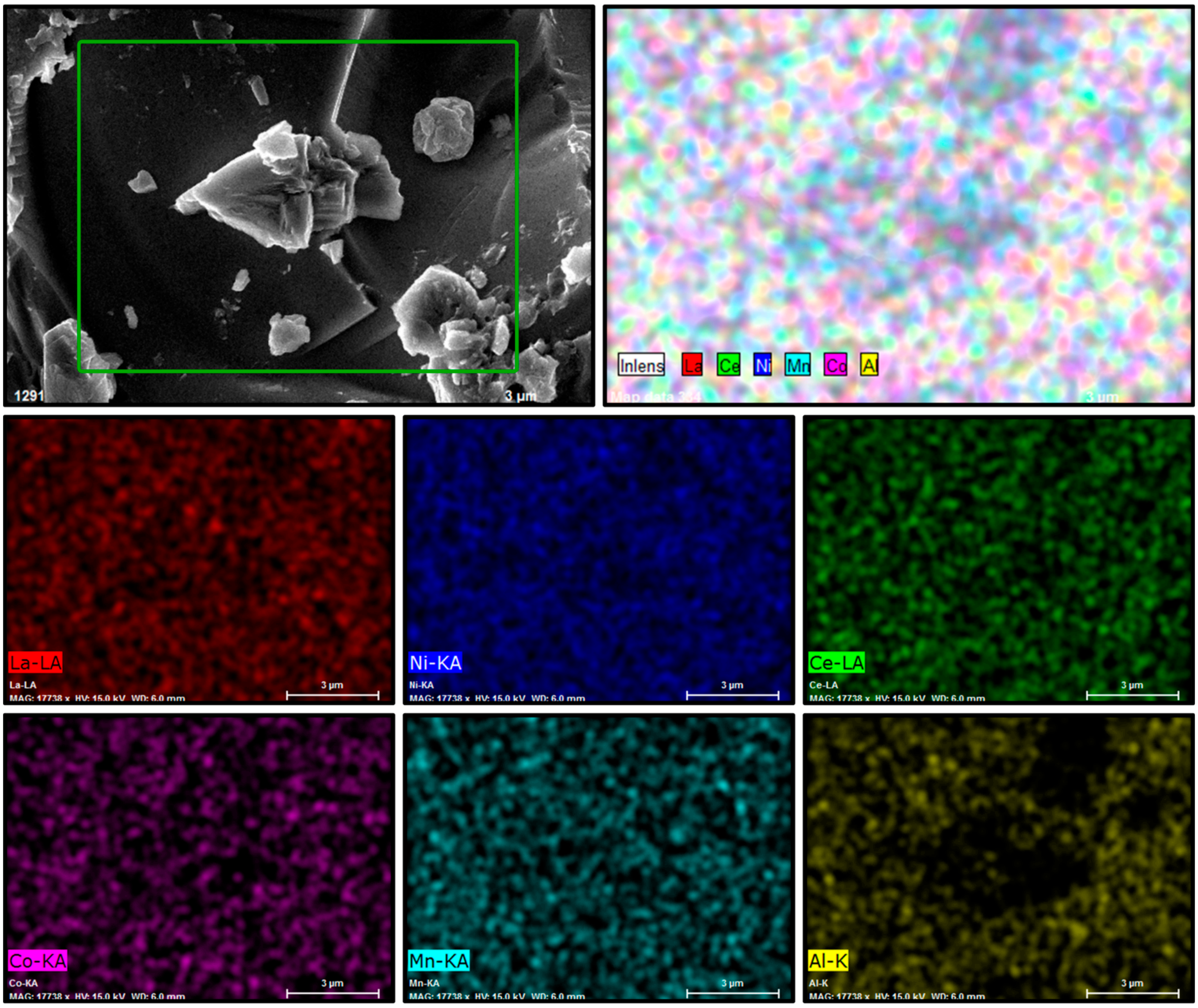

Figure S3. EDS images of a surface of pristine $LaMmNi_{4.1}Al_{0.2}Mn_{0.4}Co_{0.45}$ alloy with mapping of elemental distribution of lanthanum, nickel, cerium, cobalt, manganese and aluminium.

1. LaMm-$Ni_{4.1}Al_{0.3}Mn_{0.4}Co_{0.45}$ alloy [3/3]

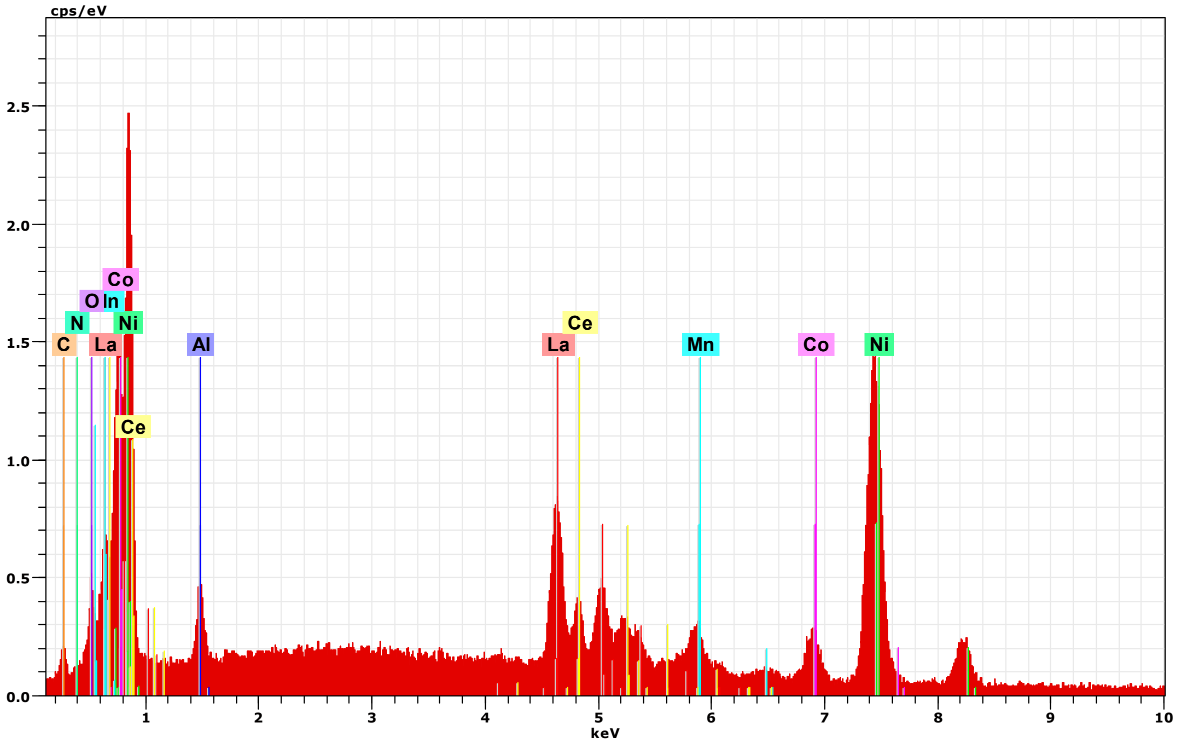

Figure S4. EDS spectrum of a surface of pristine $LaMmNi_{4.1}Al_{0.2}Mn_{0.4}Co_{0.45}$ alloy not subjected to electrochemical treatment [M. Karwowska *et al.*, *J. Power Sources*, 263 (**2014**) 304].

Table S1. Results of EDS elemental analysis of pristine $LaMmNi_{4.1}Al_{0.2}Mn_{0.4}Co_{0.45}$ alloy not subjected to electrochemical treatment [M. Karwowska *et al.*, *J. Power Sources*, 263 (**2014**) 304].

Element	Series	unn. C [wt %]	norm. C [wt %]	Atom. C [at %]	Error [wt %]
Lanthanum	L-series	19.48	19.91	8.52	0.60
Nickel	K-series	55.72	56.94	57.67	1.77
Aluminium	K-series	1.95	2.00	4.40	0.13
Manganese	K-series	3.99	4.08	4.41	0.16
Cobalt	K-series	5.62	5.75	5.80	0.23
Cerium	L-series	7.63	7.80	3.31	0.26
Carbon	K-series	2.01	2.06	10.19	0.48
Nitrogen	M-series	0.48	0.49	2.07	0.19
Oxygen	K-series	0.96	0.98	3.63	0.23
	Total:	97.85	100.00	100.00	

2. Electrochemical capacity of LaMm-$Ni_{4.1}Al_{0.3}Mn_{0.4}Co_{0.45}$ alloy in alkaline solutions

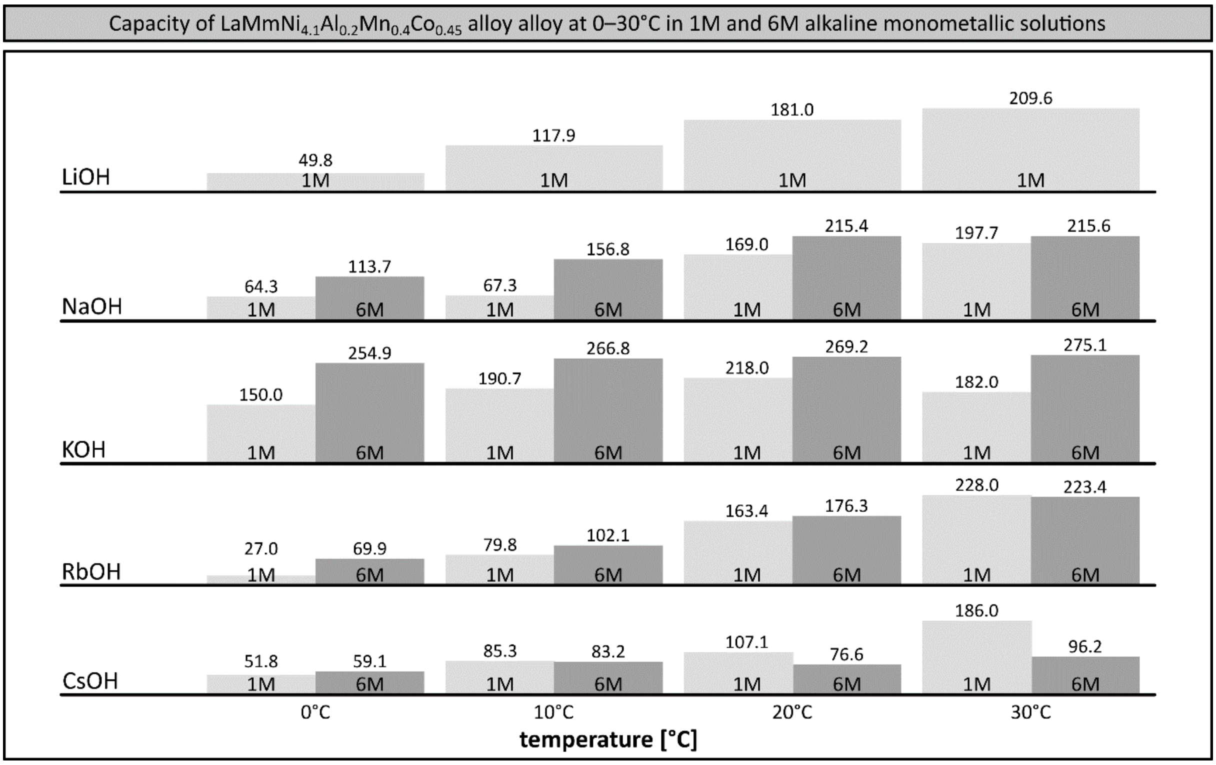

Figure S5. Electrochemical capacity of LaMm–$Ni_{4.1}Al_{0.3}Mn_{0.4}Co_{0.45}$ alloy as a function of composition of 1M and 6M MOH (M = Li,Na,K,Rb,Cs) solutions at temperatures in the range 0–30 °C in a series of decreasing temperatures [M. Karwowska *et al.*, *Electrochim. Acta.*, 252 (**2017**) 381].

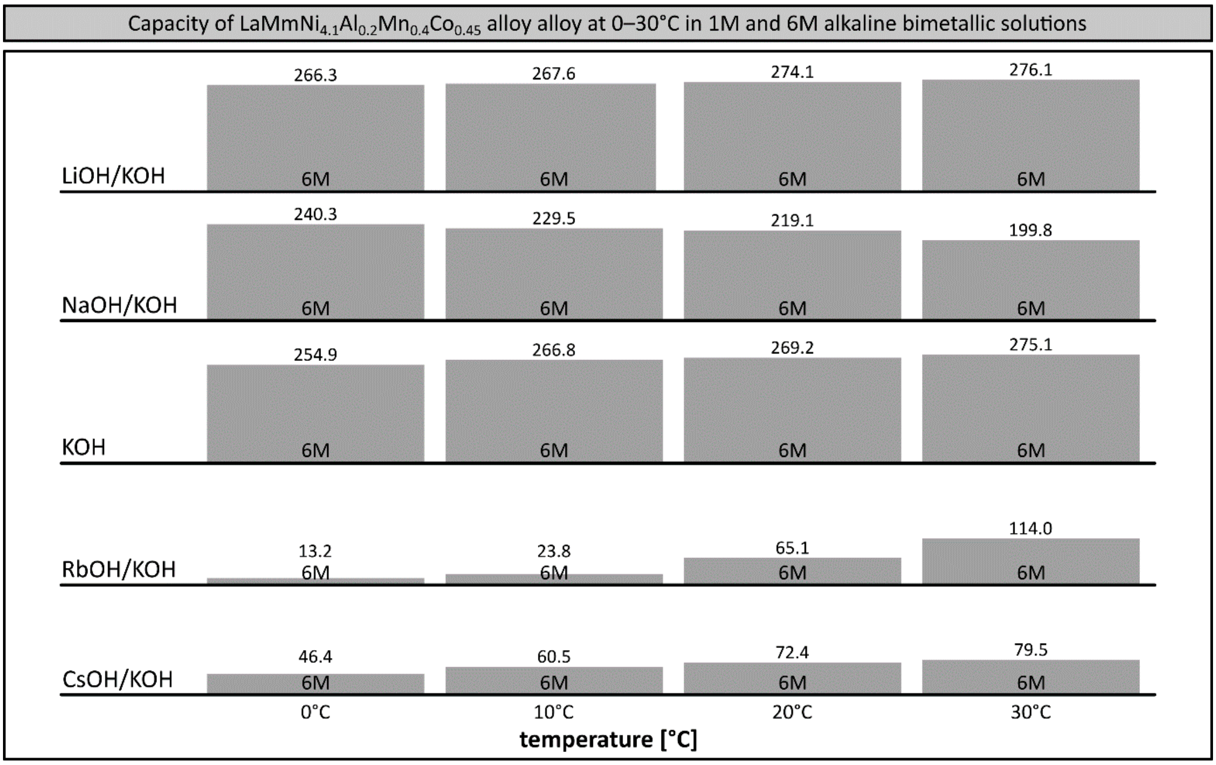

Figure S6. Electrochemical capacity of LaMm–$Ni_{4.1}Al_{0.3}Mn_{0.4}Co_{0.45}$ alloy as a function of composition of 6M MOH/KOH (M = Li–Cs) solutions at temperatures in the rang 0–30°C in a series of decreasing temperature [M. Karwowska *et al.*, *Electrochim. Acta.*, 252 (**2017**) 381].

3. Mechanical degradation of LaMm-$Ni_{4.1}Al_{0.3}Mn_{0.4}Co_{0.45}$ alloy after gaseous H_2 treatment

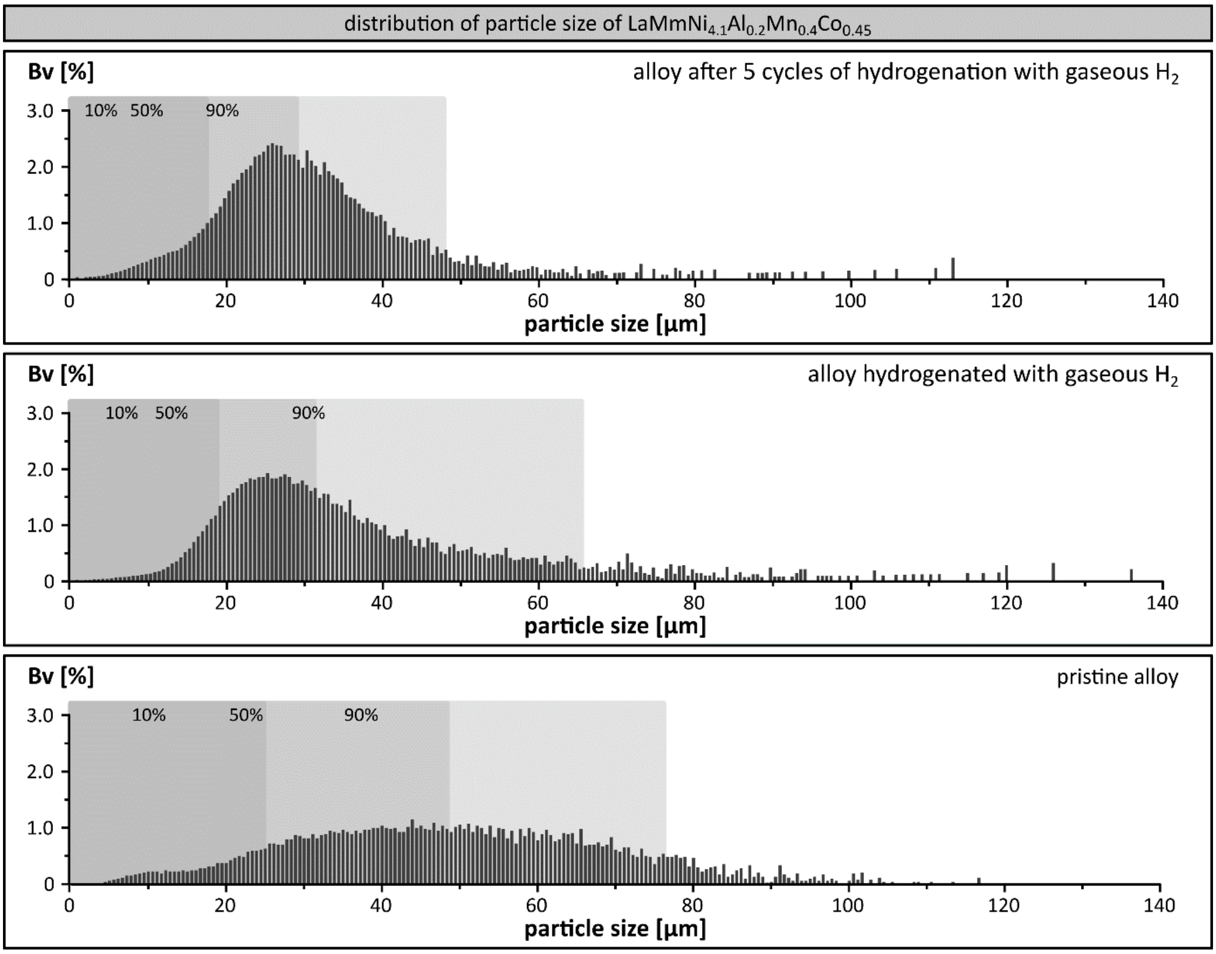

Figure S7. Distribution of the particle size of the $LaMmNi_{4.1}Al_{0.2}Mn_{0.4}Co_{0.45}$ alloy: pristine alloy (bottom), hydrogenated alloy (middle) and alloy after 5 cycles of hydrogenation with gaseous H_2 (top). Particle size [μm] showed in a function of volume fraction Bv [%]. Percentile values of 10%, 50% and 90% of the population exposed with grey fields. Sample population e 200,000 particles. Spectrum of pristine alloy were shown by us before [M. Karwowska *et al.*, *J. Power Sources*, 263 (**2014**) 304].

Table S2. Results of granulometric determination of particle size distribution of the $LaMmNi_{4.1}Al_{0.2}Mn_{0.4}Co_{0.45}$ alloy: pristine alloy, hydrogenated alloy and alloy after 5 cycles of hydrogenation with gaseous H_2. Parameters of pristine alloy were shown by us before [M. Karwowska *et al.*, *J. Power Sources*, 263 (**2014**) 304].

Parameter	Pristine alloy	Hydrogenated Alloy	Alloy after 5 cycles
Percentie 10%	24.1 μm	19.3 μm	18.0 μm
Percentie 50%	48.8 μm	32.4 μm	29.5 μm
Percentie 90%	76.7 μm	65.6 μm	47.8 μm
spherical coefficient	1.592	2.091	2.327
Specific mass surface	606 cm^2/g	772 cm^2/g	887 cm^2/g
Specific volume surface	1539 cm^2/cm^3	1961 cm^2/cm^3	2227 cm^2/cm^3

4. EDS images of surface formations observed after treatment in KOH solution

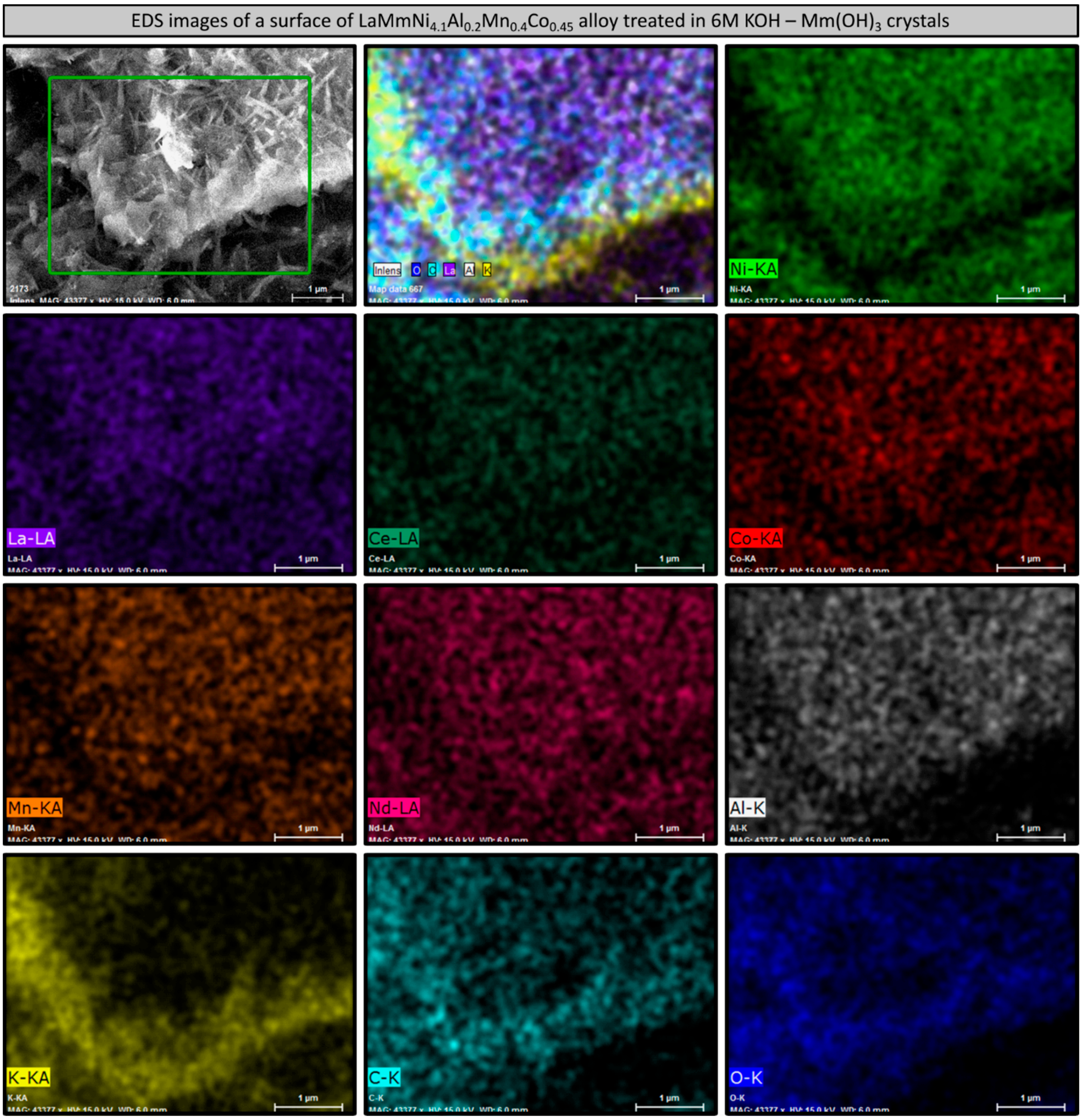

Figure S8. EDS surface images of $LaMmNi_{4.1}Al_{0.2}Mn_{0.4}Co_{0.45}$ alloy electrochemically treated in 6M KOH at 30 °C. Mapping of elemental distribution of rubidium, lanthanum, praseodymium, aluminium, nickel, manganese and cerium.

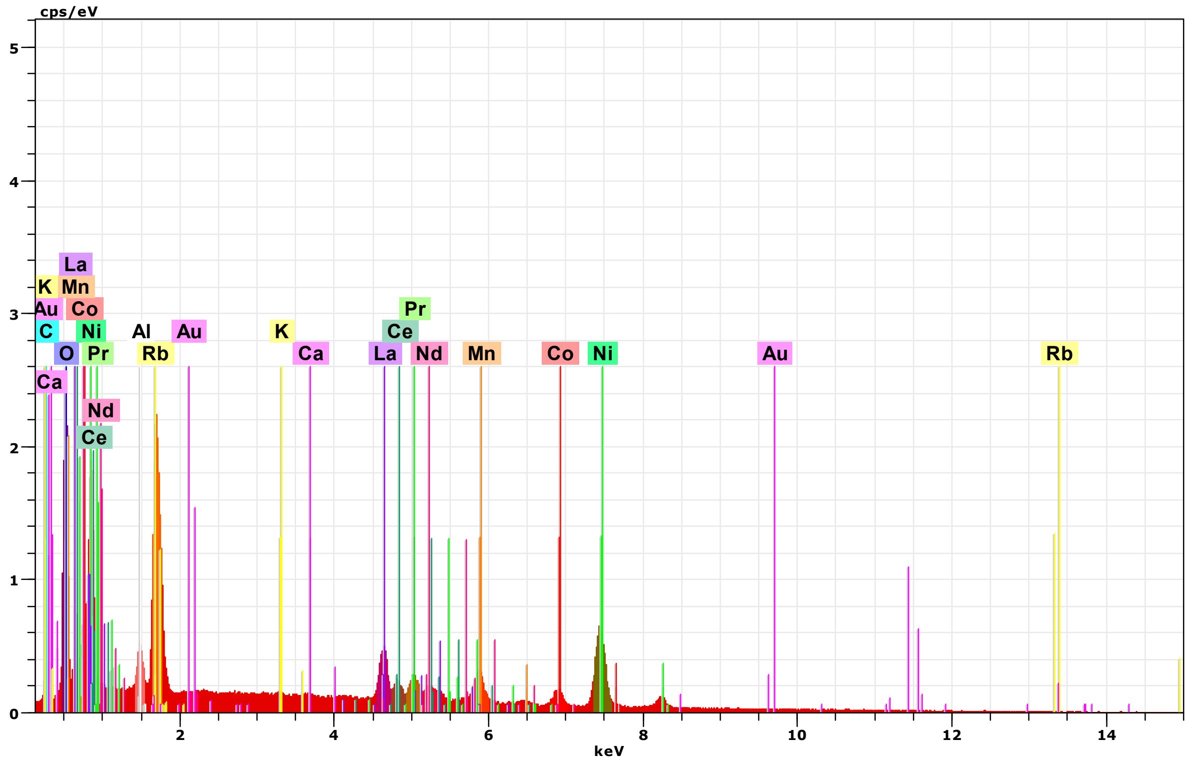

Figure S9. EDS spectrum of the corrosive layer at the surface of $LaMmNi_{4.1}Al_{0.2}Mn_{0.4}Co_{0.45}$ alloy electrochemically treated in 6M KOH at 30 °C.

Table S3. Results of EDS elemental analysis of the corrosive layer at the surface of $LaMmNi_{4.1}Al_{0.2}Mn_{0.4}Co_{0.45}$ alloy electrochemically treated in 6M KOH at 30 °C.

Element	Series	unn. C [wt %]	norm. C [wt %]	Atom. C [at %]	Error [wt %]
Oxygen	K-series	18.36	19.15	46.47	2.24
Carbon	K-series	4.13	4.31	13.92	0.73
Nickel	K-series	23.01	24.00	15.87	0.75
Cobalt	K-series	3.56	3.71	2.44	0.15
Manganese	K-series	4.60	4.80	3.39	0.17
Lanthanum	L-series	11.32	11.81	3.30	0.36
Cerium	L-series	4.42	4.61	1.28	0.16
Rubidium	L-series	22.55	23.52	10.68	0.98
Gold	M-series	0.16	0.17	0.03	0.04
Aluminium	K-series	1.15	1.20	1.73	0.08
Neodymium	L-series	1.77	1.84	0.50	0.09
Preseodymium	L-series	0.66	0.69	0.19	0.05
Potassium	K-series	0.09	0.09	0.09	0.03
Calcium	K-series	0.10	0.10	0.10	0.03
	Total:	95.88	100.00	100.00	

5. EDS images of surface formations observed after treatment in RbOH solution [1/10]

EDS images of a surface of $LaMmNi_{4.1}Al_{0.2}Mn_{0.4}Co_{0.45}$ alloy treated in 6M RbOH – nickel particle

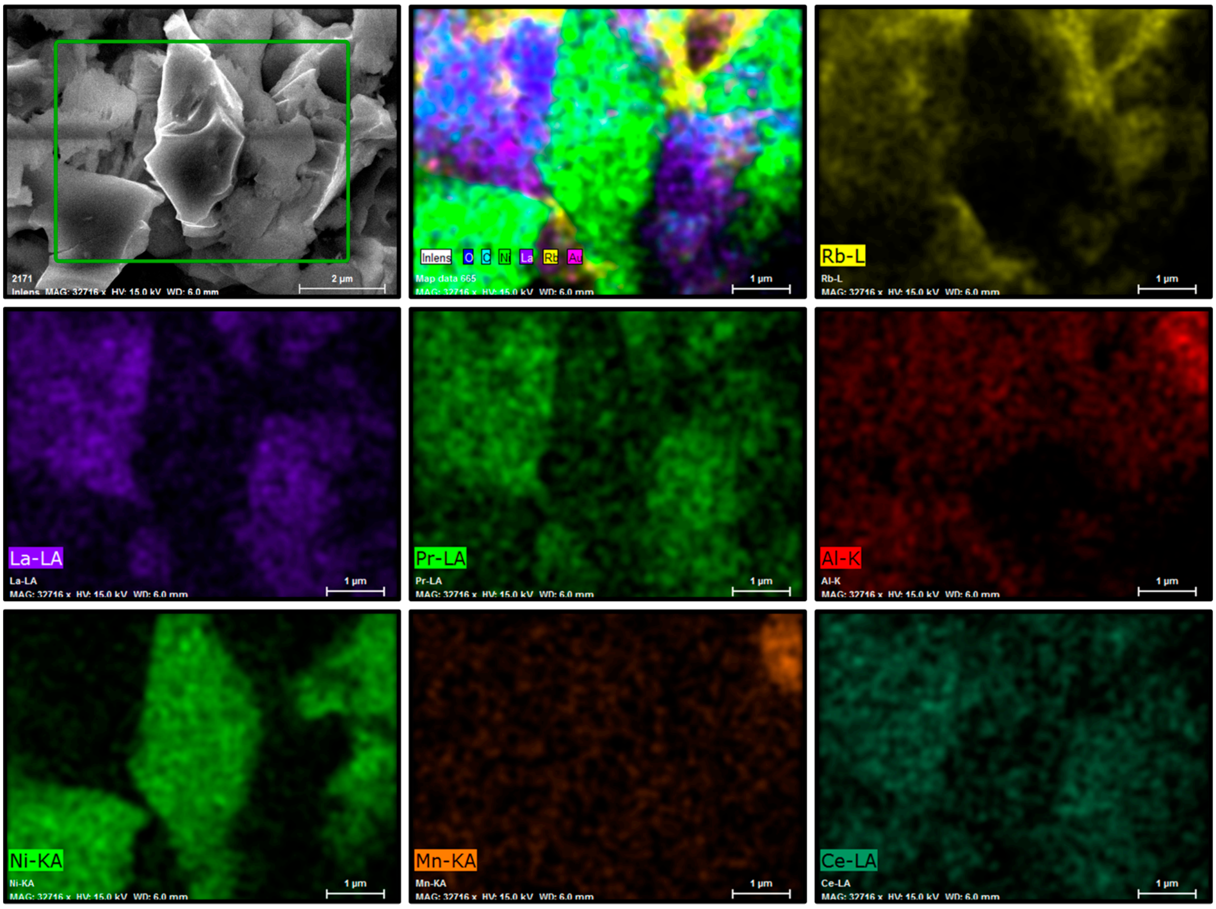

Figure S10. EDS surface images of $LaMmNi_{4.1}Al_{0.2}Mn_{0.4}Co_{0.45}$ alloy electrochemically treated in 6M RbOH at 30 °C, with visible nickel metal particle. Mapping of elemental distribution of rubidium, lanthanum, praseodymium, aluminium, nickel, manganese and cerium.

5. EDS images of surface formations observed after treatment in RbOH solution [2/10]

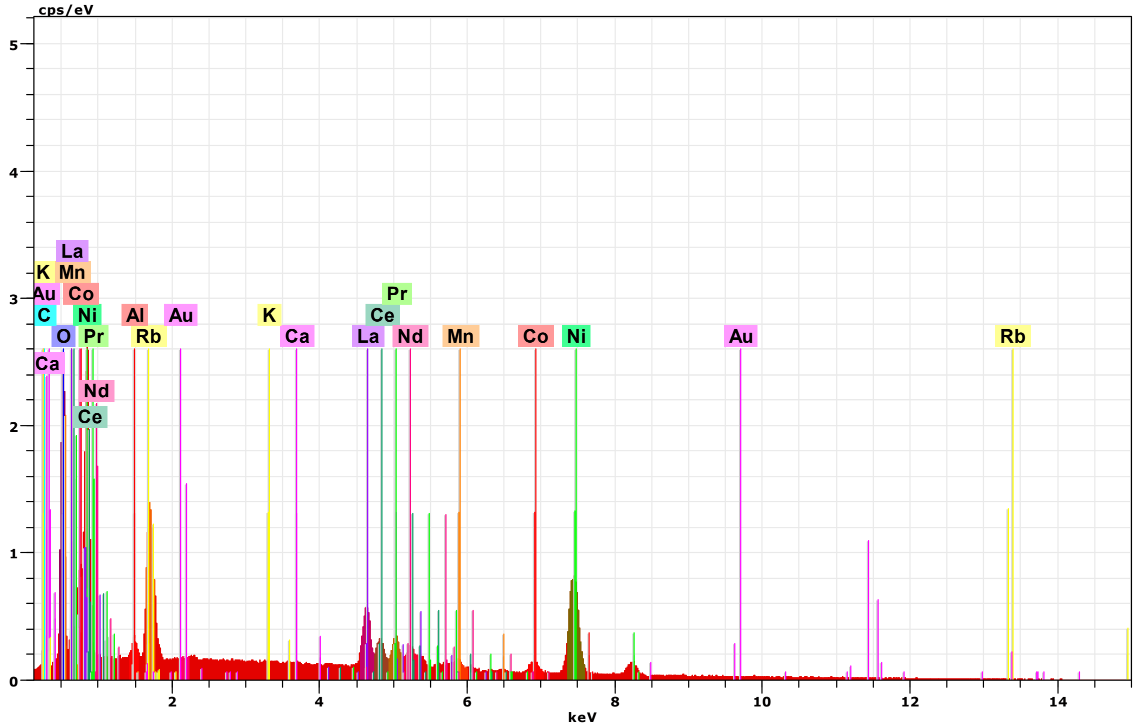

Figure S11. EDS spectrum of the area of nickel metal particle found at the surface of $LaMmNi_{4.1}Al_{0.2}Mn_{0.4}Co_{0.45}$ alloy electrochemically treated in 6M RbOH at 30 °C.

Table S4. Results of EDS elemental analysis of nickel metal particle found at the surface of $LaMmNi_{4.1}Al_{0.2}Mn_{0.4}Co_{0.45}$ alloy electrochemically treated in 6M RbOH at 30 °C.

Element	Series	unn. C [wt %]	norm. C [wt %]	Atom. C [at %]	Error [wt %]
Oxygen	K-series	15.94	16.55	44.29	1.93
Carbon	K-series	2.86	2.97	10.59	0.53
Nickel	K-series	34.03	35.34	25.78	1.08
Cobalt	K-series	2.91	3.03	2.20	0.13
Manganese	K-series	1.49	1.55	1.21	0.08
Lanthanum	L-series	14.36	14.91	4.60	0.45
Cerium	L-series	7.30	7.58	2.32	0.24
Rubidium	L-series	13.62	14.14	7.09	0.60
Gold	M-series	0.05	0.05	0.01	0.03
Aluminium	K-series	0.48	0.50	0.79	0.05
Neodymium	L-series	2.33	2.42	0.72	0.10
Prezeodymium	L-series	0.78	0.81	0.25	0.06
Potassium	K-series	0.07	0.07	0.08	0.03
Calcium	K-series	0.08	0.08	0.09	0.03
	Total:	96.31	100.00	100.00	

5. EDS images of surface formations observed after treatment in RbOH solution [3/10]

EDS images of a surface of $LaMmNi_{4.1}Al_{0.2}Mn_{0.4}Co_{0.45}$ alloy treated in 6M RbOH – La-C-O-H crystals

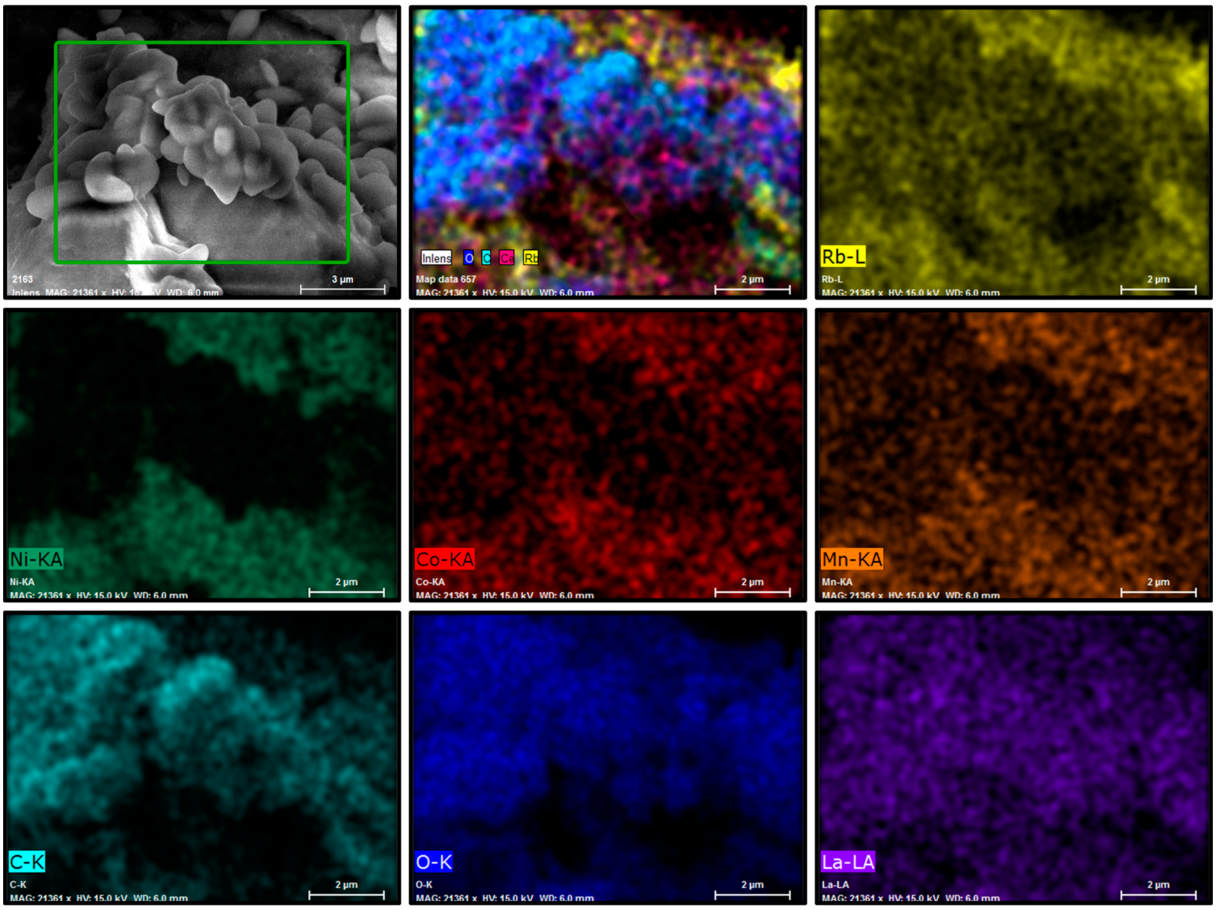

Figure S12. EDS surface images of $LaMmNi_{4.1}Al_{0.2}Mn_{0.4}Co_{0.45}$ alloy electrochemically treated in 6M RbOH at 30 °C, with visible La-C-O round-shaped crystals. Mapping of elemental distribution of rubidium, nickel, cobalt, manganese, carbon, oxygen and lanthanum.

5. EDS images of surface formations observed after treatment in RbOH solution [4/10]

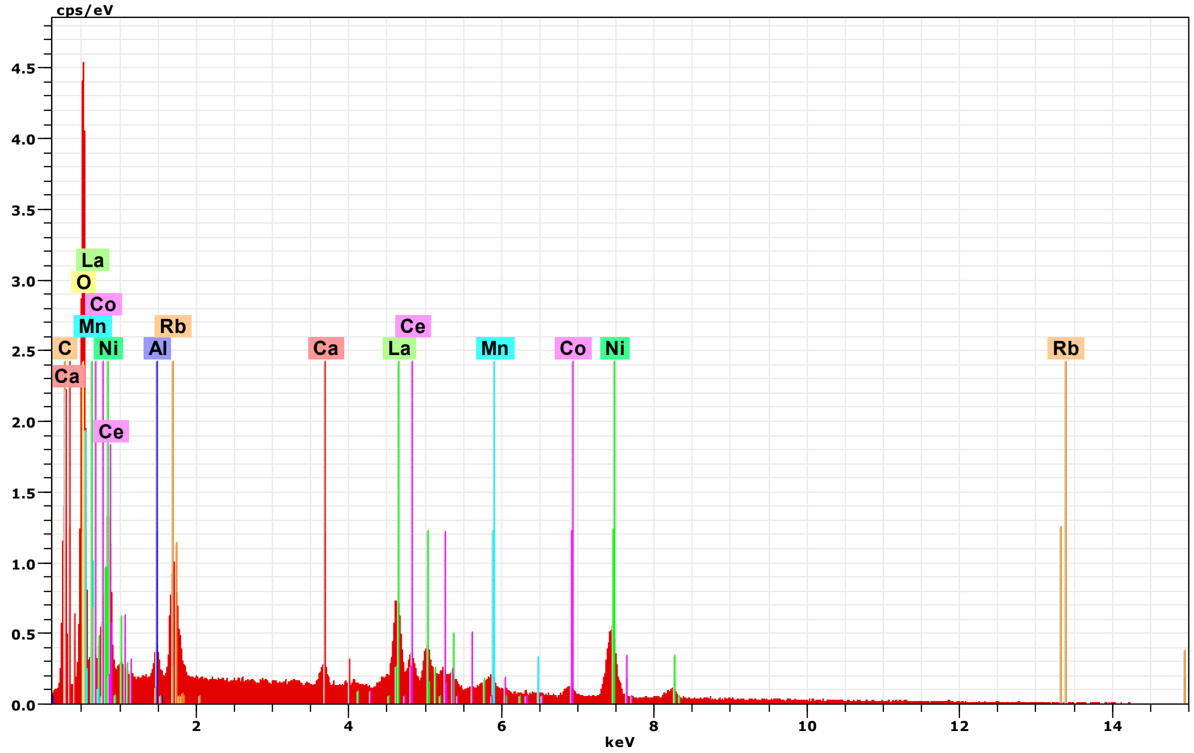

Figure S13. EDS spectrum of the area of La-C-O crystals found at the surface of $LaMmNi_{4.1}Al_{0.2}Mn_{0.4}Co_{0.45}$ alloy electrochemically treated in 6M RbOH at 30 °C.

Table S5. Results of EDS elemental analysis of the area of La-C-O crystals found at the surface of $LaMmNi_{4.1}Al_{0.2}Mn_{0.4}Co_{0.45}$ alloy electrochemically treated in 6M RbOH at 30 °C.

Element	Series	unn. C [wt %]	norm. C [wt %]	Atom. C [at %]	Error [wt %]
Nickel	K-series	19.00	19.34	9.37	0.65
Aluminium	K-series	0.52	0.53	0.56	0.06
Manganese	K-series	2.29	2.33	1.21	0.11
Cobalt	K-series	2.11	2.15	1.04	0.12
Oxygen	K-series	27.98	27.98	49.75	3.29
Carbon	K-series	12.12	12.33	29.21	1.79
Lanthanum	L-series	18.27	18.59	3.81	0.57
Cerium	L-series	7.69	7.83	1.59	0.27
Rubidium	L-series	7.50	7.63	2.54	0.35
Calcium	K-series	1.08	1.10	0.78	0.07
Potassium	K-series	0.19	0.19	0.14	0.04
	Total:	98.27	100.00	100.00	

5. EDS images of surface formations observed after treatment in RbOH solution [5/10]

EDS images of a surface of $LaMmNi_{4.1}Al_{0.2}Mn_{0.4}Co_{0.45}$ alloy treated in 6M RbOH – Rb-La-O crystals

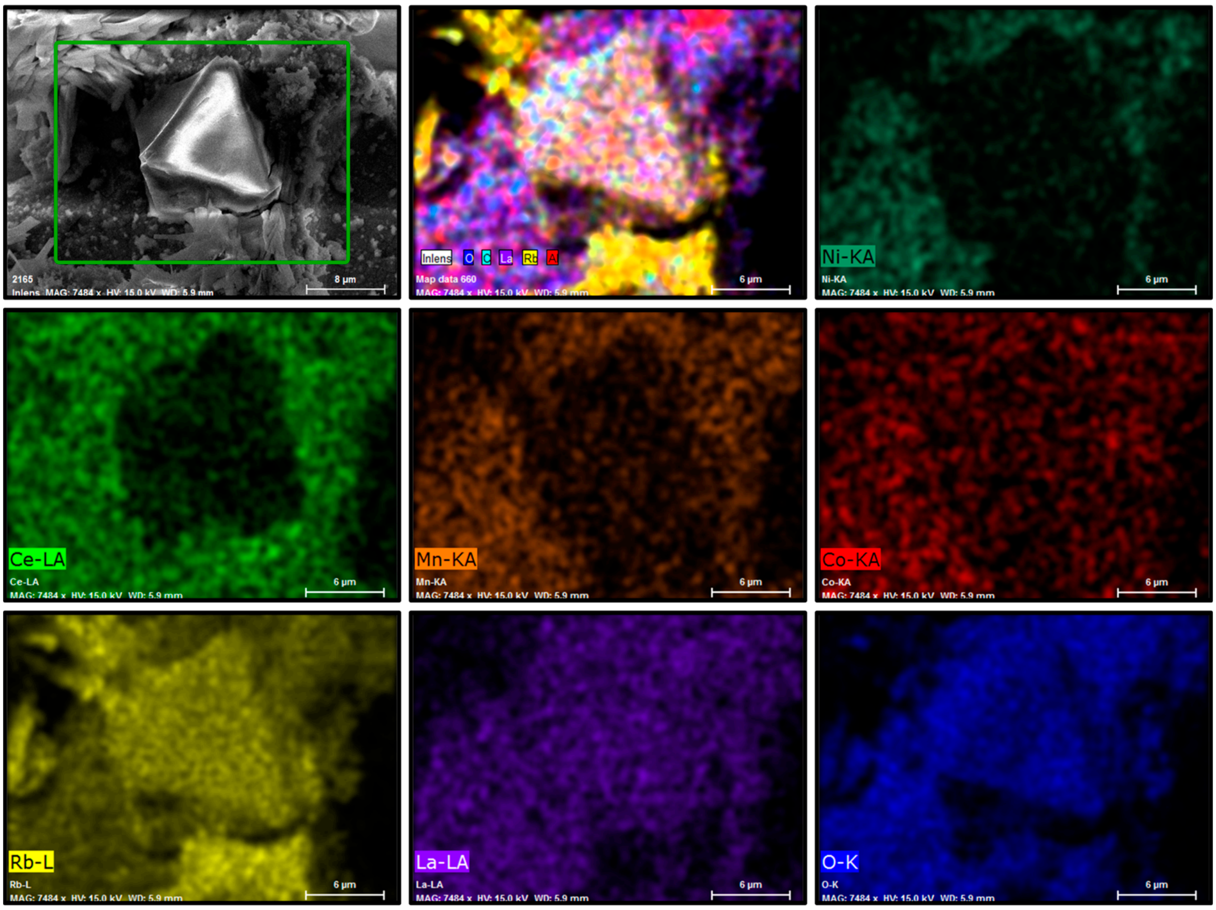

Figure S14. EDS surface images of $LaMmNi_{4.1}Al_{0.2}Mn_{0.4}Co_{0.45}$ alloy electrochemically treated in 6M RbOH at 30 °C, with visible Rb-La-O bipyramidal-shaped crystals. Mapping of elemental distribution of nickel, cerium, manganese, cobalt, rubidium, lanthanum and oxygen.

5. EDS images of surface formations observed after treatment in RbOH solution [6/10]

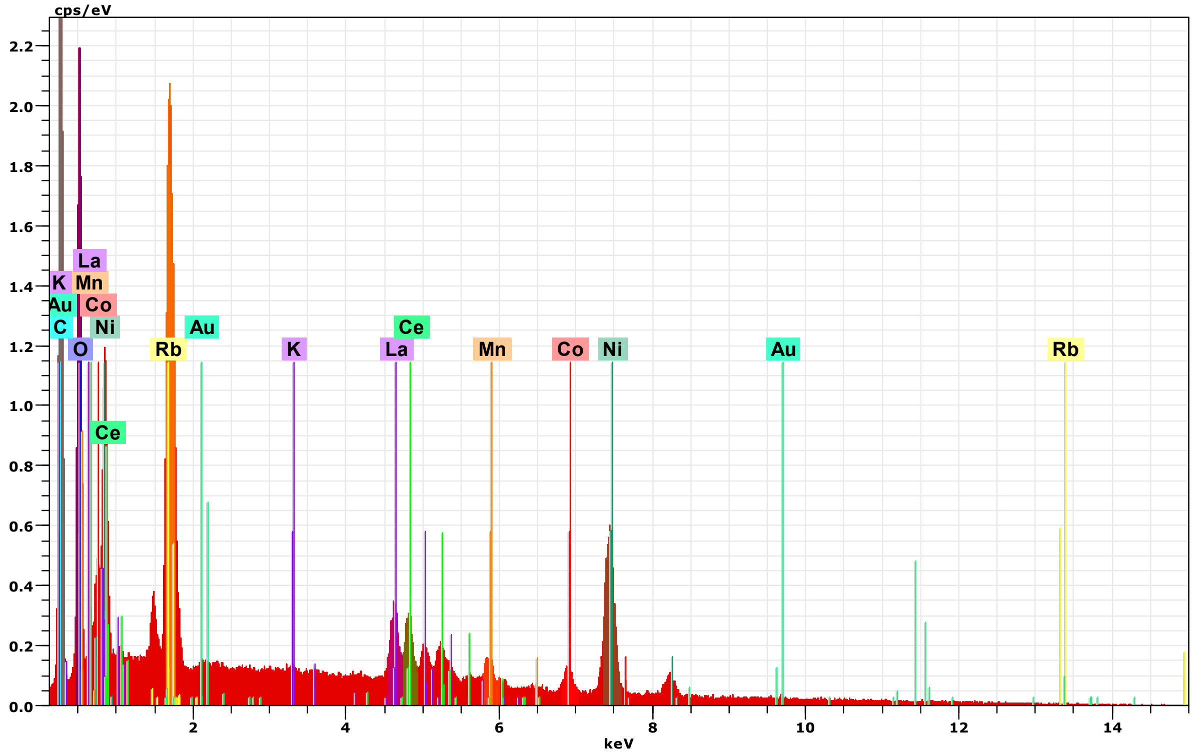

Figure S15. EDS spectrum of the area of Rb-La-O crystals found at the surface of $LaMmNi_{4.1}Al_{0.2}Mn_{0.4}Co_{0.45}$ alloy electrochemically treated in 6M RbOH at 30 °C.

Table S6. Results of EDS elemental analysis of the area of Rb-La-O crystals found at the surface of $LaMmNi_{4.1}Al_{0.2}Mn_{0.4}Co_{0.45}$ alloy electrochemically treated in 6M RbOH at 30°C.

Element	Series	unn. C [wt %]	norm. C [wt %]	Atom. C [at %]	Error [wt %]
Oxygen	K-series	18.93	17.07	24.90	2.35
Carbon	K-series	33.92	30.59	59.44	4.11
Nickel	K-series	20.00	18.04	7.17	0.65
Cobalt	K-series	2.02	1.82	0.72	0.10
Manganese	K-series	1.63	1.47	0.63	0.08
Lanthanum	L-series	7.13	6.43	1.08	0.24
Cerium	L-series	6.59	5.95	0.99	0.22
Rubidium	L-series	19.96	18.00	4.92	0.87
Potassium	K-series	0.17	0.16	0.09	0.03
Gold	M-series	0.52	0.47	0.06	0.05
	Total:	110.89	100.00	100.00	

5. EDS images of surface formations observed after treatment in RbOH solution [7/10]

EDS images of a surface of $LaMmNi_{4.1}Al_{0.2}Mn_{0.4}Co_{0.45}$ alloy treated in 6M RbOH – Rb-Ce-O crystals

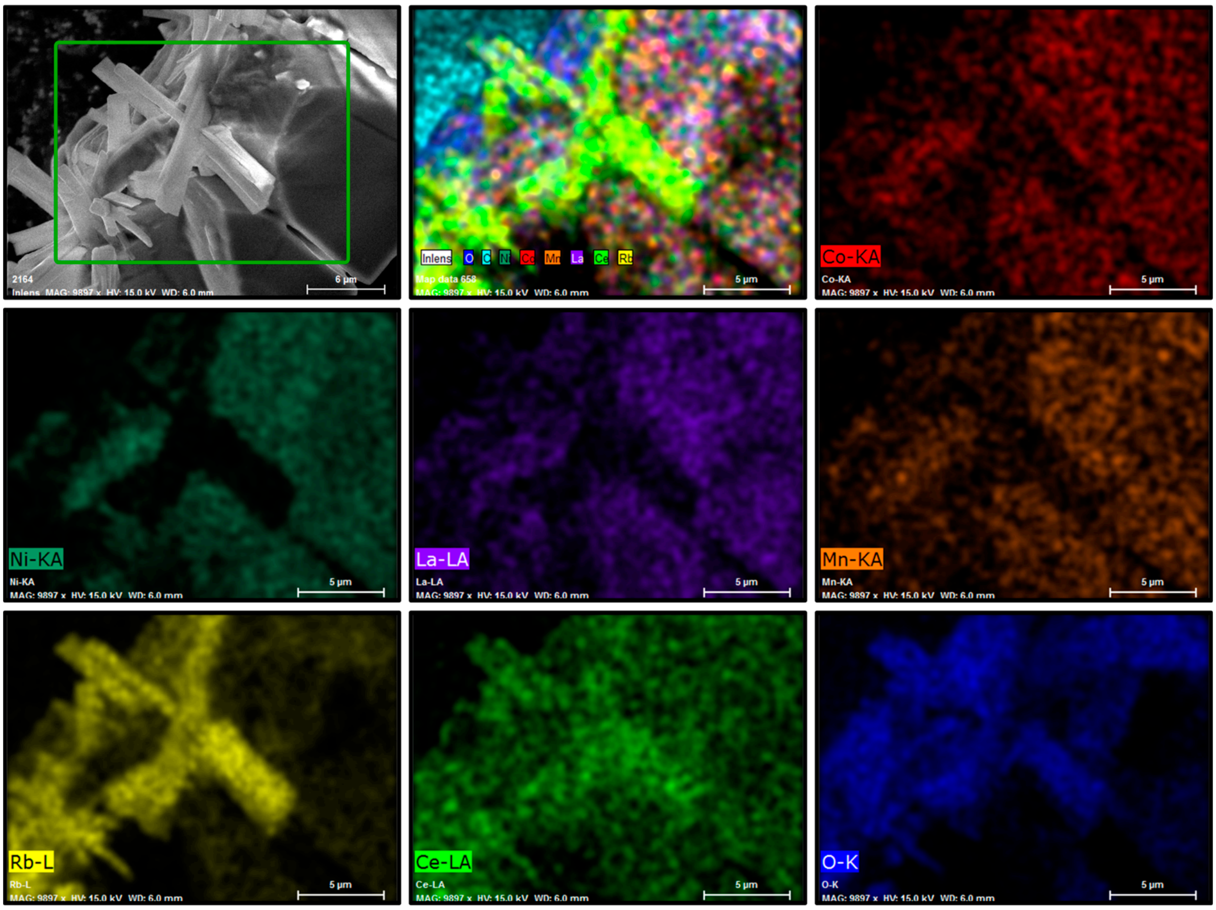

Figure S16. EDS surface images of $LaMmNi_{4.1}Al_{0.2}Mn_{0.4}Co_{0.45}$ alloy electrochemically treated in 6M RbOH at 30 °C, with visible Rb-Ce-O bipyramidal-shaped crystals. Mapping of elemental distribution of cobalt, nickel, lanthanum, manganese, rubidium, cerium and oxygen.

5. EDS images of surface formations observed after treatment in RbOH solution [8/10]

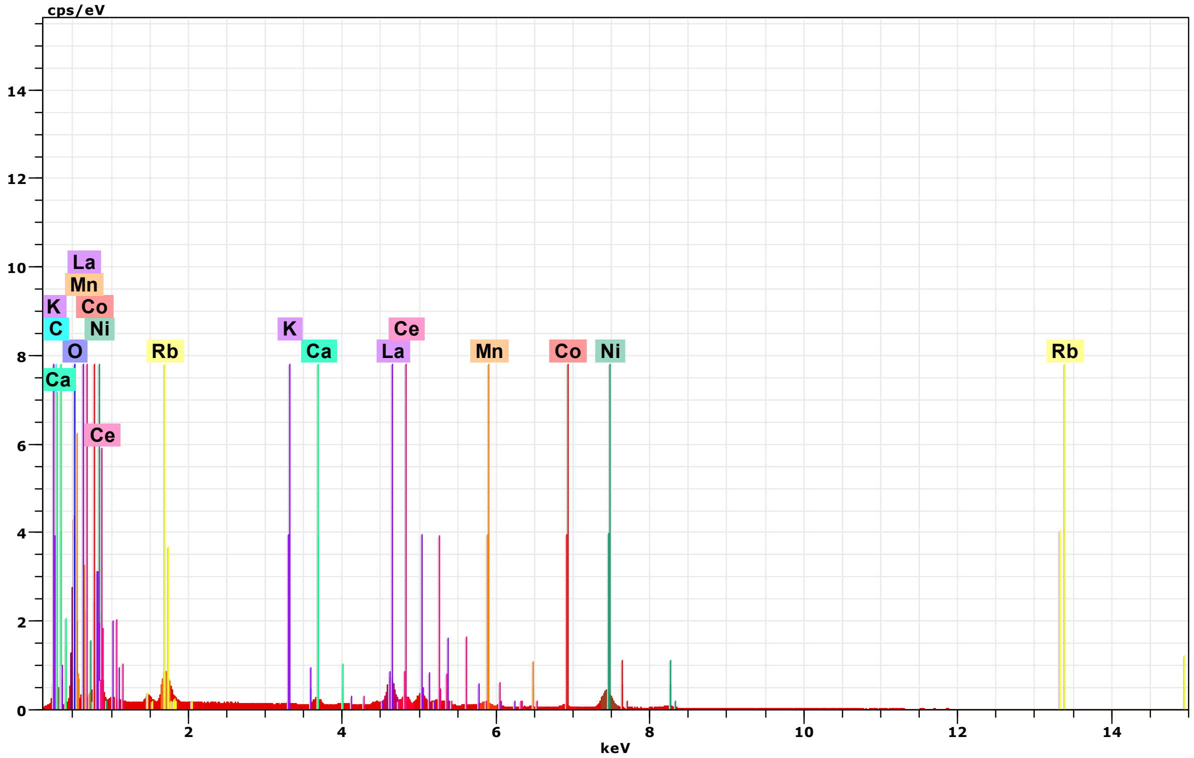

Figure S17. EDS spectrum of the area of Rb-Ce-O crystals found at the surface of $LaMmNi_{4.1}Al_{0.2}Mn_{0.4}Co_{0.45}$ alloy electrochemically treated in 6M RbOH at 30 °C.

Table S7. Results of EDS elemental analysis of the area of Rb-Ce-O crystals found at the surface of $LaMmNi_{4.1}Al_{0.2}Mn_{0.4}Co_{0.45}$ alloy electrochemically treated in 6M RbOH at 30 °C.

Element	Series	unn. C [wt %]	norm. C [wt %]	Atom. C [at %]	Error [wt %]
Oxygen	K-series	29.00	29.85	49.91	3.35
Carbon	K-series	14.10	14.51	32.32	1.83
Nickel	K-series	16.33	16.81	7.66	0.54
Cobalt	K-series	1.84	1.89	0.86	0.10
Manganese	K-series	1.93	1.99	0.97	0.09
Lanthanum	L-series	17.99	18.52	3.57	0.55
Cerium	L-series	7.55	7.77	1.48	0.25
Rubidium	L-series	6.99	7.20	2.25	0.33
Calcium	K-series	1.25	1.26	0.86	0.07
Potassium	K-series	0.17	0.17	0.12	0.03
	Total:	97.14	100.00	100.00	

5. EDS images of surface formations observed after treatment in RbOH solution [9/10]

EDS images of a surface of $LaMmNi_{4.1}Al_{0.2}Mn_{0.4}Co_{0.45}$ alloy treated in 6M RbOH – disproportionation

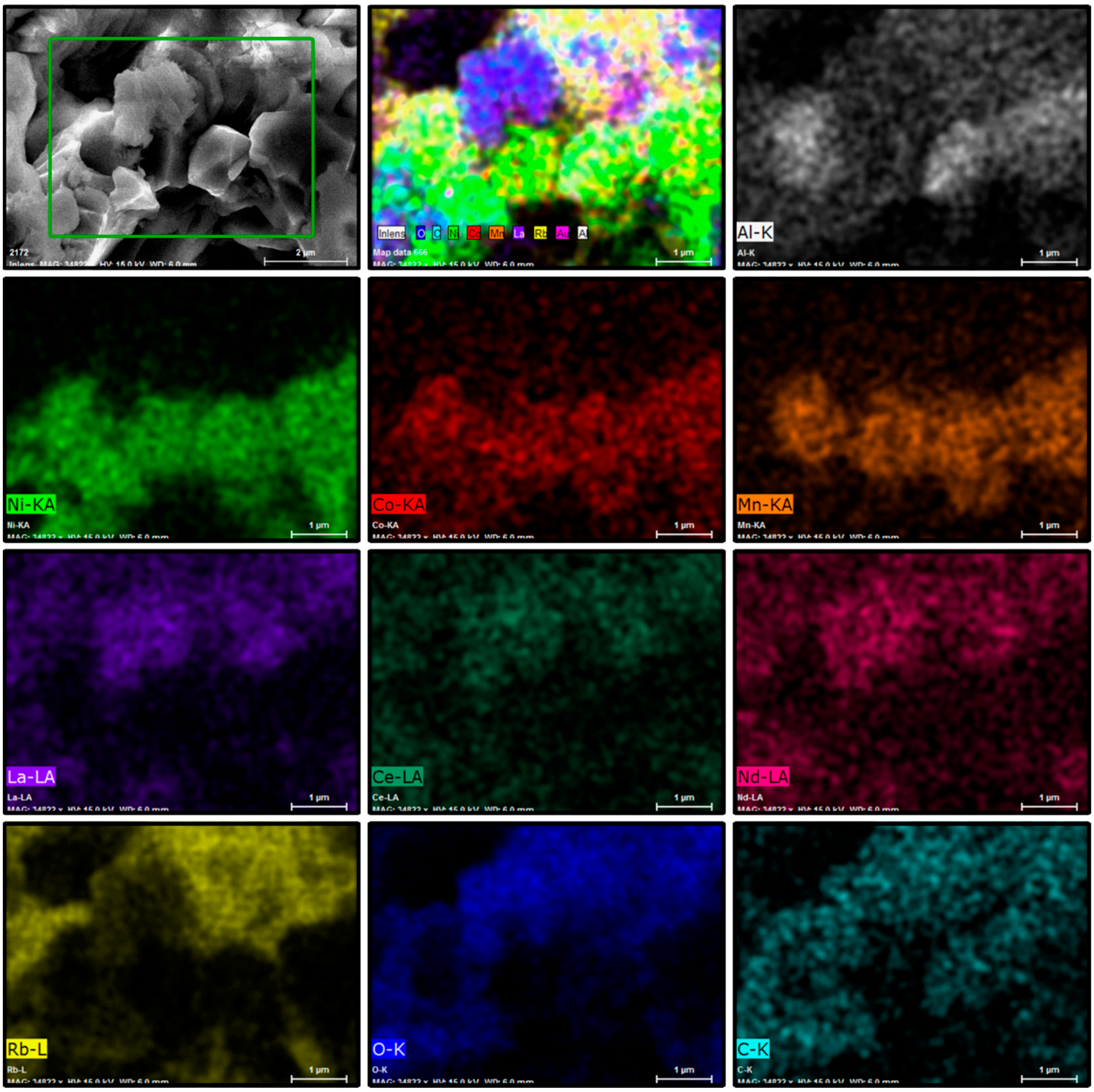

Figure S18. EDS surface images of $LaMmNi_{4.1}Al_{0.2}Mn_{0.4}Co_{0.45}$ alloy electrochemically treated in 6M RbOH at 30 °C with visible disproportionation of more-noble and less-noble elements. Mapping of elemental distribution of aluminium, nickel, cobalt, manganese, lanthanum, cerium, neodymium, rubidium, oxygen and carbon.

5. EDS images of surface formations observed after treatment in RbOH solution [10/10]

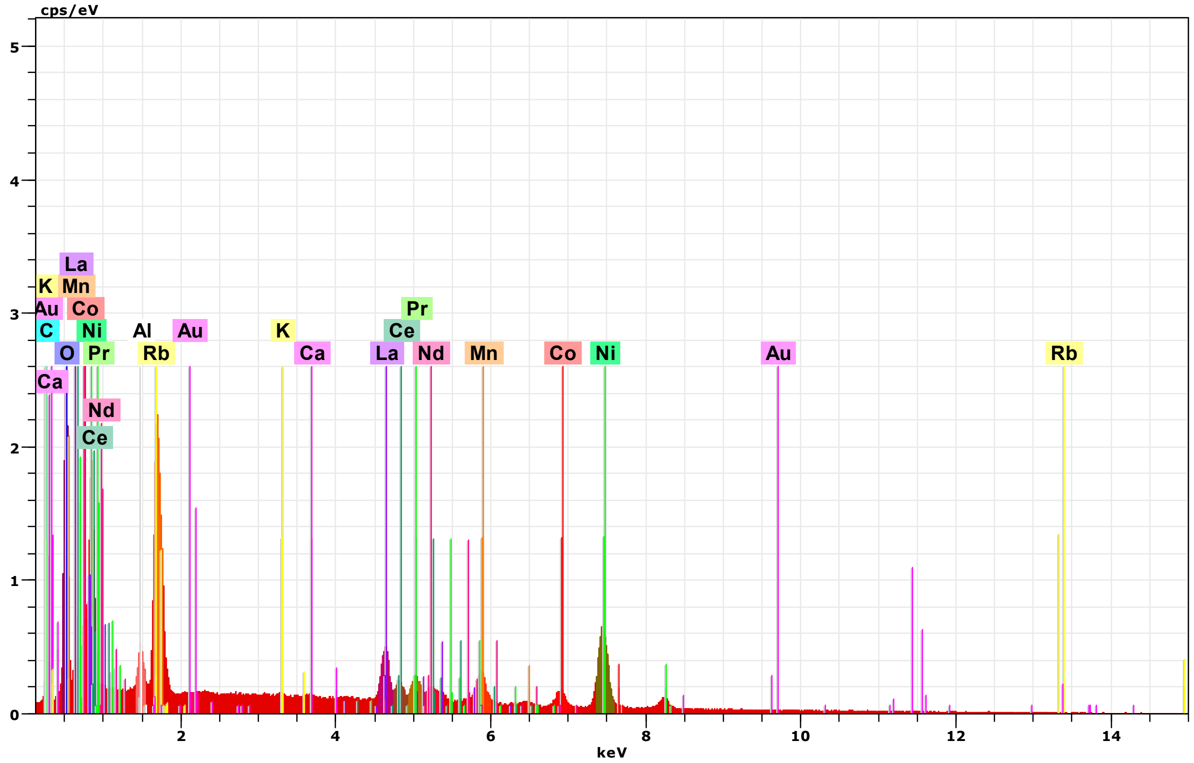

Figure S19. EDS spectrum of the surface of $LaMmNi_{4.1}Al_{0.2}Mn_{0.4}Co_{0.45}$ alloy electrochemically treated in 6M RbOH at 30 °C.

Table S8. Results of EDS elemental analysis of the surface of $LaMmNi_{4.1}Al_{0.2}Mn_{0.4}Co_{0.45}$ alloy electrochemically treated in 6M RbOH at 30 °C.

Element	Series	unn. C [wt %]	norm. C [wt %]	Atom. C [at %]	Error [wt %]
Oxygen	K-series	18.36	19.15	46.47	2.24
Carbon	K-series	4.16	4.31	13.92	0.73
Nickel	K-series	23.01	24.00	15.87	0.75
Cobalt	K-series	3.56	3.71	2.44	0.15
Manganese	K-series	4.60	4.80	3.39	0.17
Lanthanum	L-series	11.32	11.81	3.30	0.36
Cerium	L-series	4.42	4.61	1.28	0.16
Rubidium	L-series	22.55	23.52	10.68	0.98
Gold	M-series	0.16	0.17	0.03	0.04
Aluminium	K-series	1.15	1.20	1.73	0.08
Neodymium	L-series	1.77	1.84	0.50	0.09
Praseodymium	L-series	0.66	0.69	0.19	0.05
Potassium	K-series	0.09	0.09	0.09	0.03
Calcium	K-series	0.10	0.10	0.10	0.03
	Total:	95.88	100.00	100.00	

6. EDS images of surface formations observed after treatment in CsOH solution

Crystals of $CsLaO_2$ at the surface of $LaMmNi_{4.1}Al_{0.2}Mn_{0.4}Co_{0.45}$ alloy treated in 1M CsOH, and their EDS spectrum

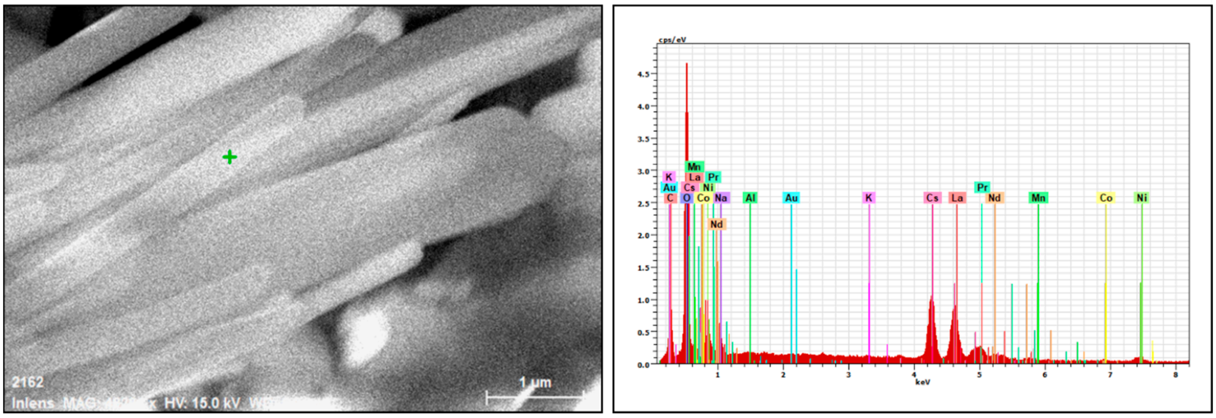

Figure S20. SEM image of Cs-La-O crystals at the surface of $LaMmNi_{4.1}Al_{0.2}Mn_{0.4}Co_{0.45}$ alloy electrochemically treated in 1M CsOH at 20 °C (left) and EDS spectrum of these crystals (right).

Table S9. Results of EDS elemental analysis of Cs-La-O crystals at the surface of $LaMmNi_{4.1}Al_{0.2}Mn_{0.4}Co_{0.45}$ alloy electrochemically treated in 1M CsOH at 20 °C.

Element	Series	unn. C [wt %]	norm. C [wt %]	Atom. C [at %]	Error [wt %]
Carbon	K-series	11.85	11.47	24.45	1.78
Oxygen	K-series	40.89	39.60	63.37	4.91
Sodium	K-series	1.80	1.75	1.95	0.15
Gold	M-series	0.18	0.17	0.02	0.04
Cesium	L-series	30.52	29.56	5.69	0.92
Nickel	K-series	3.80	3.68	1.61	0.18
Aluminium	K-series	0.11	0.11	0.11	0.03
Neodymium	L-series	1.62	1.56	0.28	0.09
Praseodymium	L-series	0.18	0.17	0.03	0.04
Lanthanum	L-series	11.47	11.11	2.05	0.38
Potassium	K-series	0.17	0.17	0.11	0.04
Cobalt	K-series	0.29	0.29	0.12	0.05
Manganese	K-series	0.17	0.17	0.08	0.04
Chlorine	K-series	0.19	0.19	0.14	0.04
	Total:	103.25	100.00	100.00	

7. Results of search in Inorganic Crystal Structure Database by FIZ Karlsruhe

Table S10. Summary of search in Inorganic Crystal Structure Database by Fachinformationszentrum Karlsruhe for known compounds comprising elements detected during EDS investigation of corrosion products observed after treatment of $LaMmNi_{4.1}Al_{0.2}Mn_{0.4}Co_{0.45}$ alloy in RbOH and CsOH. Access on 17 September 2018.

Elements searched					Records from ICSD		Reference
–	La	–	–	–	–		–
–	La	O	–	–	–		–
–	La	O	H	–	–		–
–	La	O	–	C	–		–
–	La	O	H	C	(4242-ICSD)	$La_2(CO_3)_2(OH)_2$	*Am. Mineral.* 60 (**1975**) 280.
					(22224-ICSD)	$La_2(CO_3)_3(H_2O)_8$	*Inorg. Chem.* 7 (**1968**) 1340.
					(109668-ICSD)	$La_2(C_2O_4)_3(H_2O)_{10}$	*Inorg. Nucl. Chem. Lett.* 5 (**1969**) 263.
					(109873-ICSD)	$(La(H_2O)_3)_2(C_2O_4)_3(H_2O)_4$	*J. Crystal. Spec. Res.* 21 (**1991**) 127.
					(109874-ICSD)	$(La(H_2O)_3)_2(C_2O_4)_3(H_2O)_{3.2}$	*Z. Neorg. Khimii* 45 (**2001**) 1492.
					(109875-ICSD)	$(La(H_2O)_3)_2(C_2O_4)_3(H_2O)_{3.5}$	*Mater. Res. Bull.* 23 (**1988**) 579.
					(109981-ICSD)	$(La(H_2O)_4)_2(C_4O_4)_2(C_2O_4)(H_2O)_{2.5}$	*Z. Anorg. Allg. Chem.* 627 (**2001**) 2173.
					(165652-ICSD)	$La((C_2O_4)_{0.5}(OH)_2)(H_2O)_3$	*J. Molecular Structure* 932 (**2009**) 123.
					(251980-ICSD)	$La_2(C_2O_4)_3(H_2O)_{9.5}$	*Inorg. Chem.* 54 (**2015**) 8529.
					(422732-ICSD)	$La(CO_3)(OH)$	*Z. Krist. – Cryst. Mater.* 226 (**2011**) 518.
Rb	La	–	–	–	–		–
Rb	La	O	–	–	(27331-ICSD)	$RbLaO_2$	*Z. Anorg. Allg. Chem.* 417 (**1975**) 213.
Rb	La	O	H	–	–		–
Rb	La	O	–	C	–		–
Rb	La	O	H	C	–		–
Rb	Ce	–	–	–	–		–
Rb	Ce	O	–	–	(27331-ICSD)	Rb_2CeO_3	*Z. Anorg. Allg. Chem.* 433 (**1977**) 189.
Rb	Ce	O	H	–	–		–
Rb	Ce	O	–	C	–		–
Rb	Ce	O	H	C	–		–
Cs	La	–	–	–	–		–
Cs	La	O	–	–	–		–
Cs	La	O	H	–	–		–
Cs	La	O	–	C	–		–
Cs	La	O	H	C	–		–

© 2018 by the authors. Submitted for possible open access publication under the terms and conditions of the Creative Commons Attribution (CC BY) license (http://creativecommons.org/licenses/by/4.0/).
